# Biodiminution of lithium in forest floor food webs

**DOI:** 10.1038/s41598-026-46717-1

**Published:** 2026-04-03

**Authors:** Norah Muisa, Matthew Long-Hei Cheng, Martin Tsz-Ki Tsui

**Affiliations:** 1 School of Life Sciences, The Chinese University of Hong Kong, Shatin, N.T., Hong Kong SAR, China; 2 Department of Earth and Environmental Sciences, The Chinese University of Hong Kong, Shatin, N.T., Hong Kong SAR, China; 3 State Key Laboratory of Marine Environmental Health, City University of Hong Kong, Kowloon Tong, Kowloon, Hong Kong SAR China

**Keywords:** Forest floor food chain, Earthworms, Feeding habit, Biodiminution, Non-essential elements, Ecology, Ecology, Environmental sciences

## Abstract

Lithium (Li) is an emerging contaminant of global concern due to its rapidly increasing demand for rechargeable batteries. However, concerns exist regarding its bioaccumulation and trophic transfer in natural food webs, with extremely scarce data particularly in terrestrial ecosystems. Here, we examined Li levels in soil, leaf litter and ground-dwelling invertebrates from 4 temperate forest sites across the United States (US) and 2 subtropical forest sites in Hong Kong (HK), China. Overall, we found statistical differences in Li levels between US and HK sites (*p* < 0.05) but not within regions (*p* > 0.05) while Li concentrations among invertebrates ranged from < 400 ng/g dry wt. (moths and butterflies) to ~ 9,000 ng/g dry wt. (earthworms). Indeed, biodimunition patterns (i.e., negative trophic magnification slope) were consistent among sites. Further, invertebrate classification by feeding habits revealed that the detritivorous invertebrates had the highest median Li (earthworms at 5,013 ng/g). Also, partially decomposed leaf litter retained ~ 5- to 10-fold higher Li compared to fresh litter (*p* < 0.05), implying that soil is the predominant source of Li to the food web components. Our study identifies that forest floor is a source of Li and suggests that the soils and decomposing litter can be an important source of Li to the lower forest floor food webs through differential feeding habits while Li does not exhibit any enrichment along the trophic transfer. We posit that detritivorous invertebrates are prone to chronic Li exposure, prompting them a priority for future Li ecological risk assessments in terrestrial ecosystems.

**Supplementary Information:**

The online version contains supplementary material available at 10.1038/s41598-026-46717-1.

## Introduction

Lithium (Li) is increasingly recognized as a contaminant of emerging concern due to its expanding industrial applications in clean energy technologies, high environmental mobility, bioaccumulation potential, and toxicity in selected organisms^1,3^. According to the International Energy Agency^4^, Li demand and share in clean energy technologies is predicted to quadruple by 2040. Primary anthropogenic activities (e.g., Li mining, groundwater extraction, industrial processing) account for ~ 500% Li mobilization into the human environment (soil, air and water) relative to natural sources (e.g., rock weathering)^3^^,5^, yet Li remains largely unregulated^2^^,6^, and is not effectively removed by conventional water and wastewater treatment systems^7^^,8^. Notably, regions without direct Li extraction or manufacturing may still exhibit elevated environmental Li levels in groundwater and soil^9^. Consequently, Li concentrations may increase in water and soil, posing risks of entry into food chains and potential impacts on ecosystems and human health (primary intake via water/vegetables)^3^^,10,^^11^.

While aquatic ecosystems are well-studied for trophic transfer of various elements^12^, terrestrial systems remain critically understudied^13^, more so for Li^14^. The first reported study on the trophic transfer of Li in complete natural food webs was conducted by Thibon et al.^15^ in marine ecosystems. In their study^15^, Li exhibited a consistent biodiminution pattern across geographic regions studied, and the Li concentrations varied by feeding habit. Subsequent trophic transfer studies focusing on Li have followed but also on aquatic ecosystems, and found similar biodiminishing pattern with filter-feeding aquatic species hosting highest Li concentrations^16^. In contrast, land-based Li studies have primarily focused on single trophic level, mainly soil-plant transfer including human food crops, livestock^17,23^, or laboratory-based (e.g., soil-earthworms)^14^. To the best of our knowledge, no reported study has yet characterized Li bioaccumulation patterns across a complete forest floor food web in unpolluted ecosystems.

In this work, we hypothesized that Li would also exhibit a biodimunition pattern in the forest floor food webs, and that the bioaccumulation patterns would differ by species-specific traits and geographic region. This is because, although Li is widely distributed and bioavailable in soil-plant systems^24^, it is phloem-immobile in plants and lipid-insoluble^25^, only entering cells by leveraging ion transporters and channels of similar cations, e.g., Na^+^/H^+^ exchangers^26^. Taken together, these factors would facilitate efficient excretion of Li and poor transfer along food chains. Also, Li bioaccumulations patterns in forest ecosystems would likely differ because it is established that warm, moist climatic conditions are characterized by elevated microbial activities, improved feed quality, and faster decomposition rates^27,29^. Together, these characteristics would introduce variations in foraging activity and invertebrate niches under varying climatic conditions.

Thus, this study addressed this critical knowledge gap by quantifying the transfer patterns of Li in soil–plant–invertebrate forest floor food webs across temperate and subtropical forests from the United States of America (*US*) and Hong Kong, China (*HK*), of similar minimal level of human activity, establishing a baseline for Li behavior in near-pristine terrestrial ecosystems. Our study attempted to provide an extensive dataset of distinct trophic transfer patterns from forests spanning different climatic regions, to increase relevance of the results. We aimed to (1) characterize food web structures using δ¹⁵N and δ¹³C stable isotopes; (2) determine Li bioaccumulation patterns by feeding habit; and (3) assess trophic transfer using the trophic magnification slope (TMS) and trophic magnification factor (TMF).

## Results and discussion

### Invertebrate trophic structure and energy sources

**Fig. 1:**
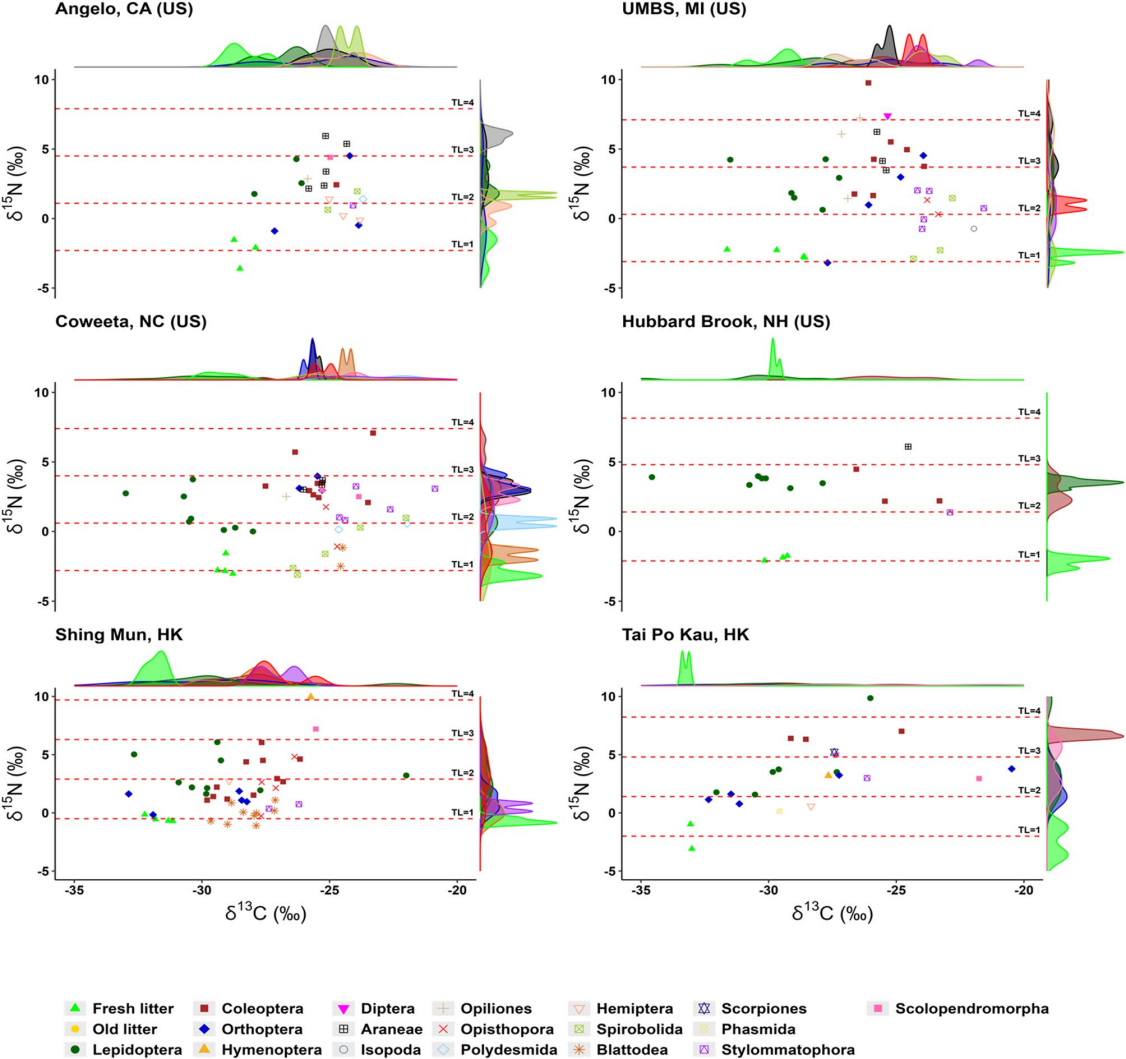
Scatter plots and density distribution plots of δ^13^C and δ^15^N of plant litter and invertebrate orders for the United States (US) and Hong Kong, China (HK) forests studied. Note that, for the order Arachnida only, the family names are used instead to differentiate harvestman, spider and scorpions. The dashed lines indicate the calculated trophic levels (TL) according to Eq. 1. It should be noted that negative stable isotopic signatures indicate depletion relative to the standard reference and vice versa. UMBS: University of Michigan Biological Station; Angelo: Angelo Coast Range Reserve; Coweeta: Coweeta LTER; Hubbard Brook: Hubbard Brook Experimental Forest; Tai Po Kau: Tai Po Kau Nature Reserve; Shing Mun: Shing Mun Country Park.

The δ^13^C signature for plant litter sampled across all the US and HK sites studied ranged from − 33‰ (HK sites only) to -29‰ (Fig. 1), typical of C_3_ plants^30^^,31^. In line with the aim of our study, the basal plant resources are similar providing comparable energy sources to their respective food chains, regardless of geographical region differences. The plant litter in HK sites were significantly depleted by ~ 3‰ than US sites (Kruskal-Wallis: *p* < 0.05), reflecting obvious climatic, edaphic and plant functional trait differences (see SI Table S1-S6)^30^^,32^. Within-region differences were insignificant (Dunn’s test: *p* > 0.05) whilst across-region differences were significant (Dunn’s test: *p* < 0.05).

The stable isotope signatures (i.e., δ^13^C and δ^15^N) of the invertebrates indicated that they are primarily supported by their local basal plant resources, as evidenced by values that either overlapped with (mostly, Lepidoptera order – butterflies and ~ 90% moths) or diverged away from the plant litter values (Fig. 1). The δ^13^C levels for the invertebrates from HK and US ranged from − 33.3‰ [Lepidoptera order (moth)] to -20.7‰ [Orthoptera order (grasshopper)] and − 34.5‰ [Lepidoptera order (moth)] to -21.0‰ [Stylommatophora order (land snail)], respectively. While animals tend to be similar in isotopic composition to their diets^30^, a 0.5 ± 0.13‰ of δ^13^C shift average in food webs, mainly a result of feed quality, but also fractionation via metabolic processes in consumers, is widely recognized^33^. However, the wide-ranging pattern towards δ^13^C-enriched levels relative to the basal plant resources and overlaps among the invertebrates cannot be wholly accounted for by carbon isotopic shifts alone (Fig. 1).

**Fig. 2:**
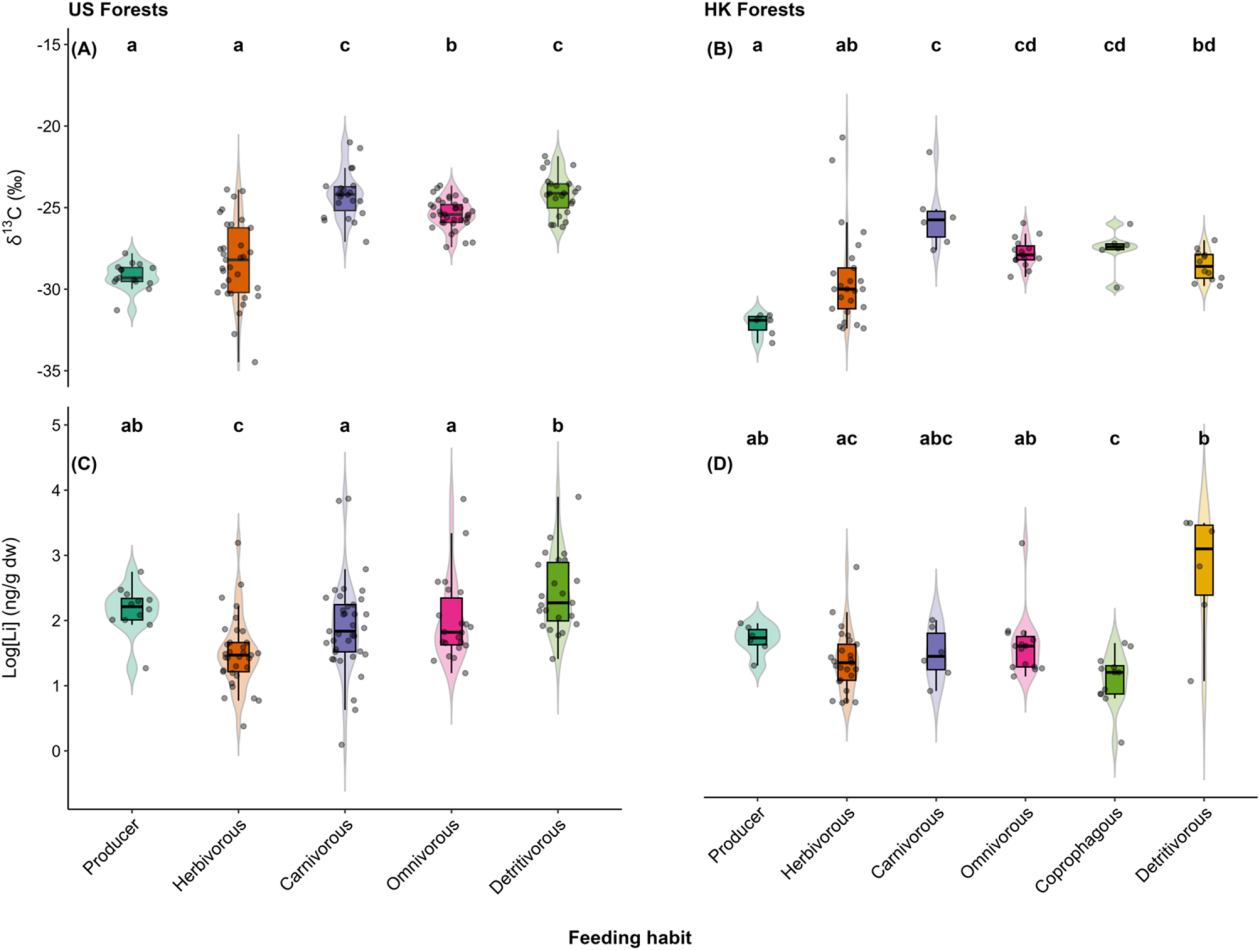
The general pattern of δ¹³C (**A** and **B**) and log_10_-transformed total Li concentrations (**C** and **D**) of the fresh leaf litter (producer) and the invertebrates categorized by their predominant feeding habits for the United States (US) and Hong Kong, China (HK) forests. Kruskal-Wallis tests revealed no evidence for significant differences in both the δ^13^C and median Li concentrations of all biota samples by locations from both [median Li: US (*p* = 0.06487) and HK (*p* = 0.5035)] regions. Medians with different letters represent significant difference in adjusted p values corresponding to 95% confidence interval (CI) using Dunn’s post hoc test with Benjamini-Hochberg correction. The boxes represent the 25th, 50th, and 75th percentile δ^13^C signatures and Li concentrations. The whiskers show the maximum and minimum. The boxes represent medians. Outliers are maintained as they reflect ecological variability.

Classifying the invertebrates by their predominant feeding habits (herbivorous, carnivorous, omnivores, detritivorous, coprophagous; SI Table S7) clarified these dietary patterns and validated the overlapping isotopic signatures (Fig. 2A), revealing patterns similar to previous findings from a tropical rainforest food web in Malaysia^34^. Our results confirm *i*) the importance and reliance of carnivores and omnivores on similar δ^13^C-enriched below-ground resources, including old leaf litter, saprophytic fungi, and detritivores^34^^,35^ depending on relative abundance and complexity of prey^36^; and *ii*) the role of omnivory, opportunistic and generalist feeding strategies among invertebrates^37^, which may blur the subsequent trophic transfer patterns.

In contrast to the US forest sites, the herbivores in HK subtropical sites also exhibited a polyphagous diet as indicated by the δ^13^C signatures that showed similarity to the signatures of both producers and detritivores (Dunn’s test: *p* > 0.05; Fig. 2B). Polyphagous diet is possible depending on food abundance, availability, succulence, physical and chemical attributes, also incorporating δ^13^C-enriched materials like fungi, old leaf litter, excreta, and woody material^34^^,35,^^38,^^39^. In fact, the warm, humid subtropical climates can significantly improve feed quality of woody material allowing for increased foraging by insects^28^. Nonetheless, the distinct δ^13^C signature of the herbivorous orders overlapping with plant litter confirms their trophic position as primary consumers, which validates them as reliable indicators of Li transfer along a soil-plant-primary consumer continuum, particularly in the US forest sites (Figs. 1 and 2A).

### Lithium distribution in the soils and plant litter

**Fig. 3:**
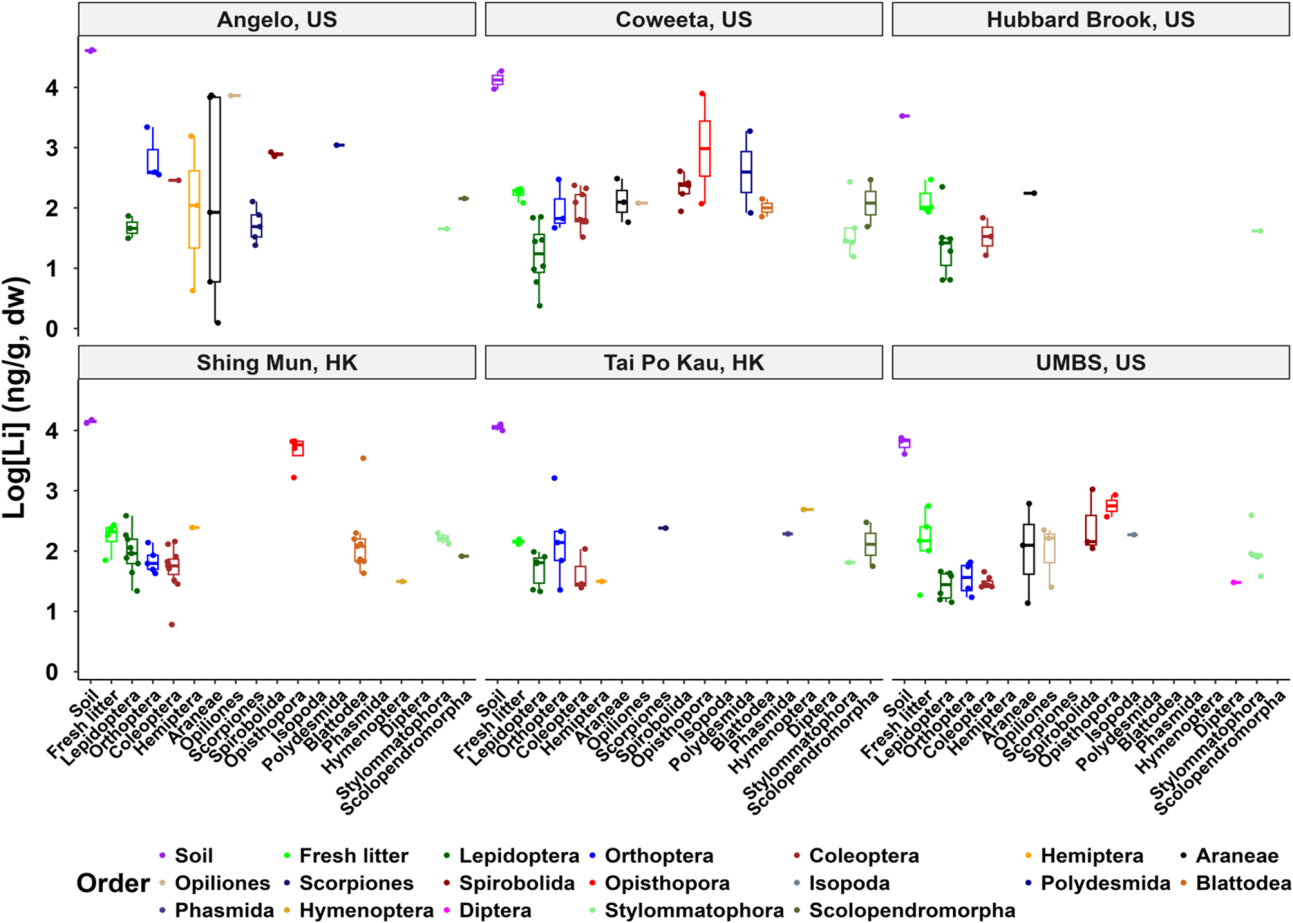
The distribution of log_10_[Li] (ng/g dry weight basis) for the soil, plant litter, and invertebrate orders, across the United States (US) and Hong Kong, China (HK) forests. Note that Orders with no values are those that were absent from the samples collected in that forest. UMBS: University of Michigan Biological Station; Angelo: Angelo Coast Range Reserve; Coweeta: Coweeta LTER; Hubbard Brook: Hubbard Brook Experimental Forest; Tai Po Kau: Tai Po Kau Nature Reserve; Shing Mun: Shing Mun Country Park.

Across the US and HK forest sites, the Li content of soil was the highest, fluctuating within a single order of magnitude (median: 8,464 ng/g- US; 12,973 ng/g- HK) apart from Angelo (mean: 40,791 ± 1,426 ng/g, *n* = 2) (Fig. 3). Generally, the basal plant resources in this study are exposed to relatively low to moderate soil Li levels based on the global soil Li average of 25,000 ng/g (Shahzad et al., 2016), with potential for uptake by plants.

The fresh leaf litter samples across all the studied sites were an order of magnitude lower (medians: 163.7 ng/g- US; 170.3 ng/g- HK) relative to source soils, with the single sample from Angelo recording an exceptionally high of 4,497 ng/g (Fig. 3). Shahzad et al.^40^ reported an earlier study by Borovik-Romanova^41^ on 138 plants from eight soil types, which established that the average Li content of terrestrial plants ranges between 150 and 300 ng/g. Further, all locations registered bioaccumulation factors (BAFs) (calculated as ratio of Li in plant to soil) < 1, confirming the low bioavailability of geogenic Li^24^, consistent with previous studies in both agricultural and forest soils^9^^,20,^^23^, and other non-essential metals (e.g., Cd, Pb)^42^. To infer on the influence of δ^13^C-enriched material feeding habit typical of detritivores, we also sampled old leaf litter (O_e_) from the same sites as fresh litter for the HK sites only. Old leaf litter significantly retained Li concentrations about 5- to 10-fold high (median: 946.4 ng/g) compared to fresh litter (Kruskal-Wallis: *p* < 0.01). Although previous Li-based studies also showed that the oldest leaves host the highest Li, they were laboratory experiments, representing different incomparable Li accumulation/retention mechanisms to field decomposing litter. It should be noted that the subsequent quantification of TL, TMS, and TMF were based only on the fresh litter results, for consistency with the US samples. Lithium accumulation in the old leaf litter could result from either leaf mass loss with C and N releases during microbial decomposition^34^^,43^, or prolonged exposure for metal exchange to occur with the soil^44^^,45^.

### Lithium bioaccumulation and trophic transfer patterns among invertebrates

Our results demonstrated in several ways that Li bioaccumulation patterns in terrestrial ecosystems are primarily governed by the interplay between the diet (specifically, leaf litter age), species trait (including growth stage and feeding habit), and to a lesser extent, by the geographic location (significant differences between temperate vs. subtropical sites – Kruskal-Wallis: *p* < 0.05 – were only between Hubbard Brook, US, against either Angelo, US or Shing Mun, HK – Dunn’s post hoc test *p* < 0.05 – likely due to small sample size in the former).

**Fig. 4:**
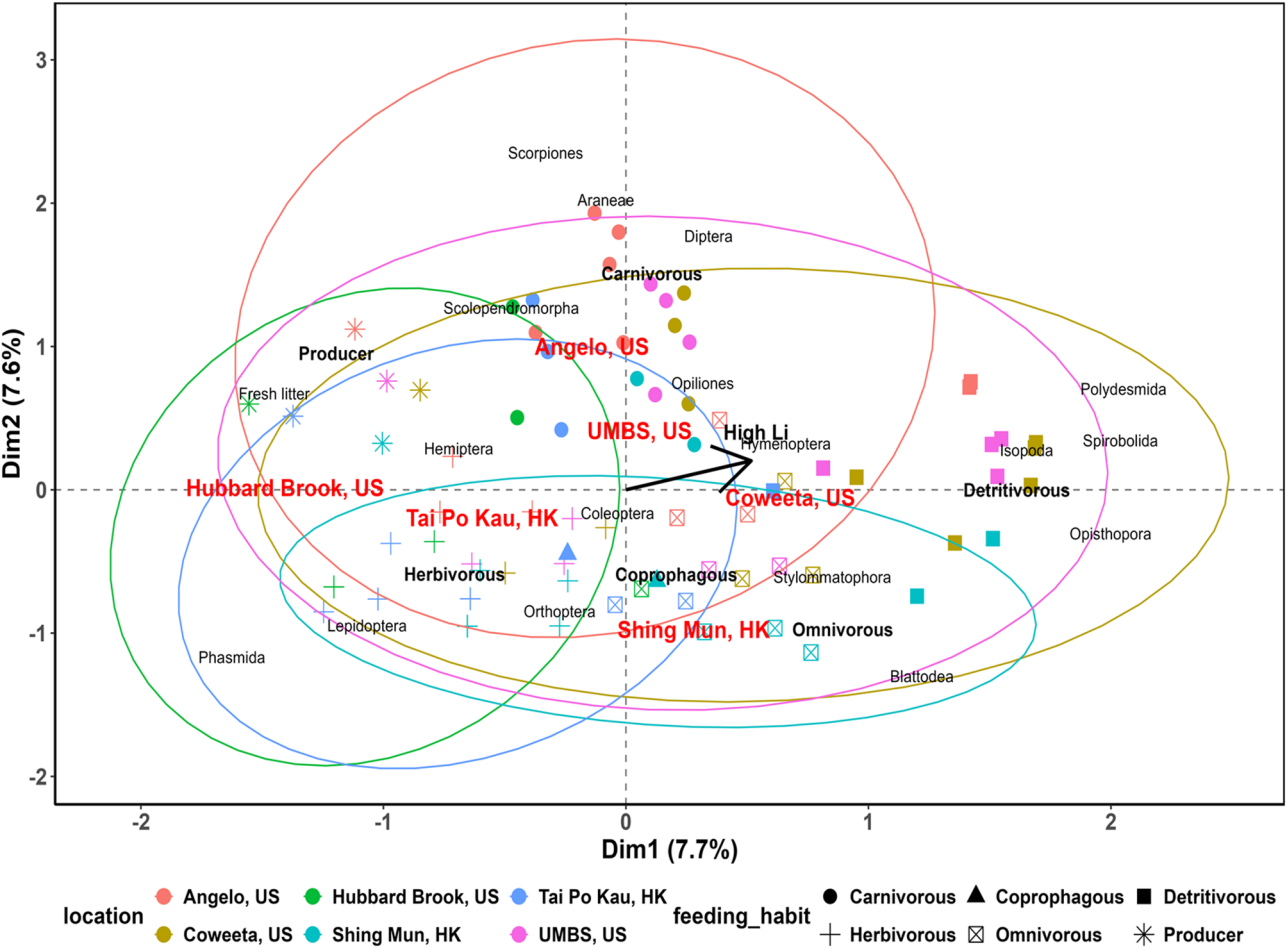
The multiple correspondence analysis (MCA) biplot results on the whole dataset (individual study forest summarized separately in SI Table S1-S6) grouped by location (i.e., forests). Other qualitative variables used are feeding habits and invertebrate order, with log_10_-transformed Li being the supplementary quantitative variable. The black arrow denotes the gradient toward groups with increasing Li levels. In contrast, coordinates of groups positioned directly opposite of the arrow – specifically in the negative portions of both dimension (dim) 1 and 2 such as the herbivores and Tai Po Kau (HK) forest – represent lower Li levels. The multiple symbols represent the individual coordinates in the space for the 200 individual samples represented in this plot colored according to the forest they originate from. UMBS: University of Michigan Biological Station; Angelo: Angelo Coast Range Reserve; Coweeta: Coweeta LTER; Hubbard Brook: Hubbard Brook Experimental Forest; Tai Po Kau: Tai Po Kau Nature Reserve; Shing Mun: Shing Mun Country Park.

First, by the MCA results we performed whereby variables occurring in closer proximity to each other in the space of the dimensions (Fig. 4) indicate close similarity. In this case, Angelo, Coweeta, and UMBS (US forests) were grouped closer to each other indicating similarities, excluding Hubbard Brook, US which instead, overlapped with Tai Po Kau, HK - a subtropical forest - suggesting the lesser impact of climatic region to Li variability among the forests. It is noteworthy that Hubbard Brook, US had the lowest total soil Li levels among all the forests (Fig. 3). Conversely, variables positioned on opposite sides of the axes and/or further away from each other indicate distinct contrasting patterns. For instance, herbivores (lower Li) *vs.* producers (higher Li levels) or herbivores and producers (low Li levels) *vs.* detritivores (high Li levels) as shown in Fig. 2B and Fig. 3. The black arrow provides the direction of coordinates associated with higher Li levels and behind it, lower levels. Also, the further away the coordinates are from the origin the stronger their contribution to the dimension. The coordinates of the categories and individuals are arranged in the first two dimensions – in this case, dimension 1 and 2 explaining a combined roughly 15% variance out of a total of 27 dimensions – relative to the average origin. Thus, detritivorous feeding habit and its specific orders (Opisthopora, Spirobolida, Polydesmida, and Isopoda) are situated furthest from the origin (Fig. 4) in agreement with their high Li levels as depicted in Figs. 2 and 3. In contrast, fresh litter and producers, as well as herbivorous (Lepidoptera order) feeding habit, are moderately further away from the origin given that Lepidoptera and fresh litter typically hosted one of the least levels of total Li (Figs. 2, D, and 3).

Second, by the tight coupling and consistent diminishing trend exhibited in both the δ^13^C signatures and Li concentrations from fresh leaf litter to the herbivorous (mainly, Lepidoptera and Orthoptera orders) feeding guild – evident in both the US and HK sites – providing a clear soil-plant-primary consumer Li transfer pattern for the first time (Fig. 2). Also, the earlier identified wide-ranging and overlapping δ^13^C signatures among non-herbivorous invertebrates (Figs. 1, 2A and 2B) is consistent with the wide variability and blurred trends across all sites in their Li concentrations (Figs. 2C, D and 3), as widely acknowledged for other metals^37^. Intraspecies differences in metal accumulation are common among invertebrates^46^^,47^.

Third, by the order of median Li concentrations by feeding habits corresponding to leaf litter total Li content: detritivorous > carnivorous ~ omnivorous > herbivorous > coprophagous invertebrates [coprophagous beetles: 29.5 ng/g (US sites); 80.5 ng/g (HK)] (Figs. 2C and 2D). Nonetheless, linear regression of stable δ^13^C revealed no significant association (*p* > 0.05) with the total Li levels of the individual invertebrates. Spearman correlation analysis also indicated weak relationships between Li, stable δ^13^C, and other parameters analyzed (SI Fig. S1). The detritivorous organisms, which predominantly feed on detritus, persistently recorded the highest Li levels (Figs. 2C and D; Table 1), corresponding to the higher Li content of old leaf litter compared to fresh leaf litter, although further study is required to ascertain this potential linkage. In particular, earthworms (order: Opisthopora) (median: 5,012.8 ng/g) contributed a 40% share among the 9% of invertebrates that exceeded the 1,000 ng Li/g threshold, based on old leaf litter highest Li levels (Table 1). However, these Li levels are within the range obtained for other non-essential elements in earthworms from uncontaminated sites^48^^,49^, and far below the median lethal concentration (LC50) of ~ 95,000 ng/g for soil Li^14^. Other orders with high median Li levels included Spirobolida and Polydesmida (millipedes), Opiliones (harvestman), Araneae (spiders) (Table 1). Interestingly, besides earthworms, millipedes and spiders are already regarded as macro-concentrators of other non-essential elements, such as Cd^48,50,51^. Noteworthy, about 95% invertebrates with > 1,000 ng/g Li are either carnivorous, omnivorous or detritivorous in line with previous patterns in δ^13^C signatures highlighted in earlier section (Table 1). Under natural field conditions, detritivorous invertebrates and their predators are prone to chronic and acute Li exposure, making them a priority for future Li ecological risk assessments in more impacted terrestrial ecosystems.

**Table 1:** List of plant litter and the 9 % of the invertebrates that accumulated greater than 1,000 ng/g of total Li concentrations on a dry weight basis from the study forests in the United States (*US*) and Hong Kong, China (*HK*), based on the old leaf litter threshold ( N.D.= not determined; N.A. = not applicable). UMBS: University of Michigan Biological Station; Angelo: Angelo Coast Range Reserve; Coweeta: Coweeta LTER.

Location	Order	Common name	Feeding habit	*n*	Total Li (ng/g)	Log_10_[Li] (ng/g)
UMBS, US	Spirobolida	Millipede	Detritivorous	N.D.	1,059.7	3.03
Angelo, US	Leaf litter	N.A.	N.A.	1	4,496.7	3.65
Angelo, US	Orthoptera	Cricket	Omnivorous	4	2,191.1	3.34
Angelo, US	Arachnida	Harvestman	Omnivorous	32	7,301.9	3.86
Angelo, US	Arachnida	Spider	Carnivorous	95	7,393.6	3.87
Angelo, US	Arachnida	Spider	Carnivorous	84	6,842.8	3.84
Angelo, US	Hemiptera	Plant bug	Herbivorous	35	1,550.9	3.19
Angelo, US	Polydesmida	Millipede	Detritivorous	17	1,100.0	3.04
Coweeta, US	Polydesmida	Millipede	Detritivorous	5	1,875.9	3.27
Coweeta, US	Opisthopora	Earthworm	Detritivorous	1	7,891.2	3.89
Tai Po Kau, HK	Orthoptera	Grasshopper	Herbivorous	1	1,623.1	3.21
Shing Mun, HK	Blattodea	Cockroach	Omnivorous	2	3,469.4	3.54
Shing Mun, HK	Opisthopora	Earthworm	Detritivorous	2	6,580.9	3.82
Shing Mun, HK	Opisthopora	Earthworm	Detritivorous	2	6,629.4	3.82
Shing Mun, HK	Opisthopora	Earthworm	Detritivorous	2	1,659.6	3.22
Shing Mun, HK	Opisthopora	Earthworm	Detritivorous	2	5,066.9	3.71

Fourth, notable was the high Li levels in the moth larval stages (up to ~ 350 ng/g) relative to those in the adults ( < ~ 100 ng/g) of the same Geometrid moth species from Coweeta, US and Shing Mun, HK, where moth larvae were also encountered and collected (SI Table S3 and SI Table S6, respectively), suggesting that growth stage is a likely factor influencing Li levels in invertebrates as reported in a meta-analysis study by Kraus et al.^52^. In fact, an adult moth sample had Li concentration below the detection limit of 3.10 ± 2.45 ng/g. We excluded larval data from all analyses to standardize the reported results to adult growth phases only. Differences in nutritional demands and mouthparts drive dietary shifts between juvenile and adult stages^39^^,53,^^54^. This pattern, compounded by Li-specific properties (i.e., phloem-immobility and accumulation in leaves)^20^^,55^, can explain the higher Li in geometrid moth larvae (caterpillar) than in the adult forms. We hypothesize that the main diet of adults consisting of sugar-rich, readily accessible fluids and soft foods like nectar, is likely ‘Li-poor’ compared to protein-rich fresh leaves consumed by the larval caterpillar stages. Another plausible explanation is body mass loss differences during metamorphosis^52^. According to Van Huis^56^, lepidopterans constitute the highest consumed edible insect globally, particularly caterpillar worms (larvae), posing an exposure risk to predators, including humans, which warrants further study in contaminated sites. The influence of growth stage on Li body burdens of invertebrates also warrants further study. The incorporation of Li and compound-specific stable nitrogen isotope analysis, as well as DNA metabarcoding techniques in future studies could assist in streamlining the Li sources and pathways.

### Overall trophic transfer pattern of Li

**Fig. 5:**
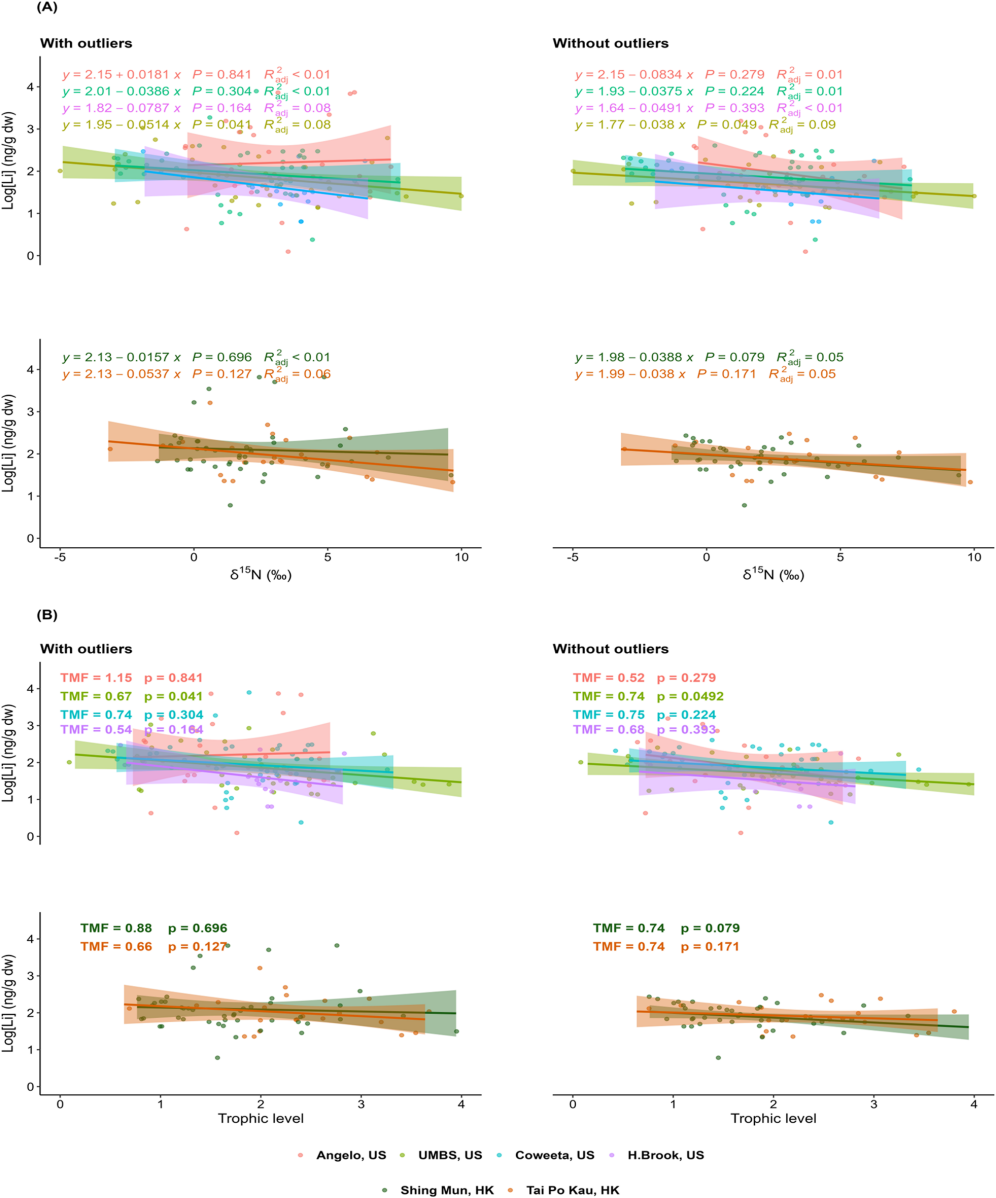
Linear regression of log_10_ [Li] against (**A**) stable nitrogen isotope composition, δ^15^N (‰), and (**B**) calculated trophic levels (TL), either with or without the identified outliers (SI Table S8). The shaded region represents the 95% confidence band of the fitted curves. UMBS: University of Michigan Biological Station; Angelo: Angelo Coast Range Reserve; Coweeta: Coweeta LTER; H. Brook: Hubbard Brook Experimental Forest; Tai Po Kau: Tai Po Kau Nature Reserve; Shing Mun: Shing Mun Country Park.

While all sites registered TMS < 0 and TMF < 1 – indicating trophic biodimunition – this pattern was only statistically significant (*p* < 0.05) in UMBS (US) forest, with or without the invertebrates identified as outliers (~ 14% of sample population; SI Table S8) (Fig. 5). This overall diminishing pattern is in line with the Li food web studies from aquatic systems^15,16^and is expected given Li’s lipid-insolubility^25^, and low (~ 80) covalent index^57^. The poor binding capacity of Li limits increases its chances of getting lost across food chains^52^^,57^. The negative TMS values for Li are in stark contrast to the significant biomagnification of methylmercury (MeHg) (TMS: 0.202 to 0.281; *p* < 0.001) identified from the same US forest ecosystems on the same invertebrate samples already reported^58^. This disparity accentuates the biodimunition tendency of Li in forest floor food webs.

Outliers could greatly affect the TMS/TMF outcomes for Li in the studied forest floor food webs. For instance, removing the outliers revealed the concealed trophic diminishing pattern in Angelo (US); the TMS/TMF models from Tai Po Kau (HK) became more obvious, whilst the models became statistically weaker in UMBS, revealing the impact of outliers on the regression models (Fig. 5; SI Table S8). These outliers were dominated by detritivores, carnivores and omnivores (SI Table S8). However, removing all outliers did not exert as much effect as removing earthworms alone (although we have not shown the no-earthworms scenario), as earthworms generally had the highest Li levels for the same trophic level (SI Table S9), highlighting the importance of dietary source in Li transfer rather than trophic position. We infer that the detritus-based invertebrates counteracted the fresh litter-based pathways, hence weakening the significance of the biodimunition patterns.

We also found that enriched δ^13^C signatures did not always translate to high Li contents as evidenced by the coprophagous beetles (Fig. 2). On the other hand, depleted δ^13^C values frequently coincided with reduced Li concentrations, e.g., Lepidoptera and Orthoptera herbivorous orders. Although coprophagous beetles, which depend on herbivorous dung^59^, share similarly enriched C signature as detritivores (Fig. 2A), herbivorous animals mainly forage fresh leaves and grass which has significantly lower Li compared to old leaf litter (and likely, soil organic matter) on which detritivores depend upon.

### Environmental implications

**Fig. 6:**
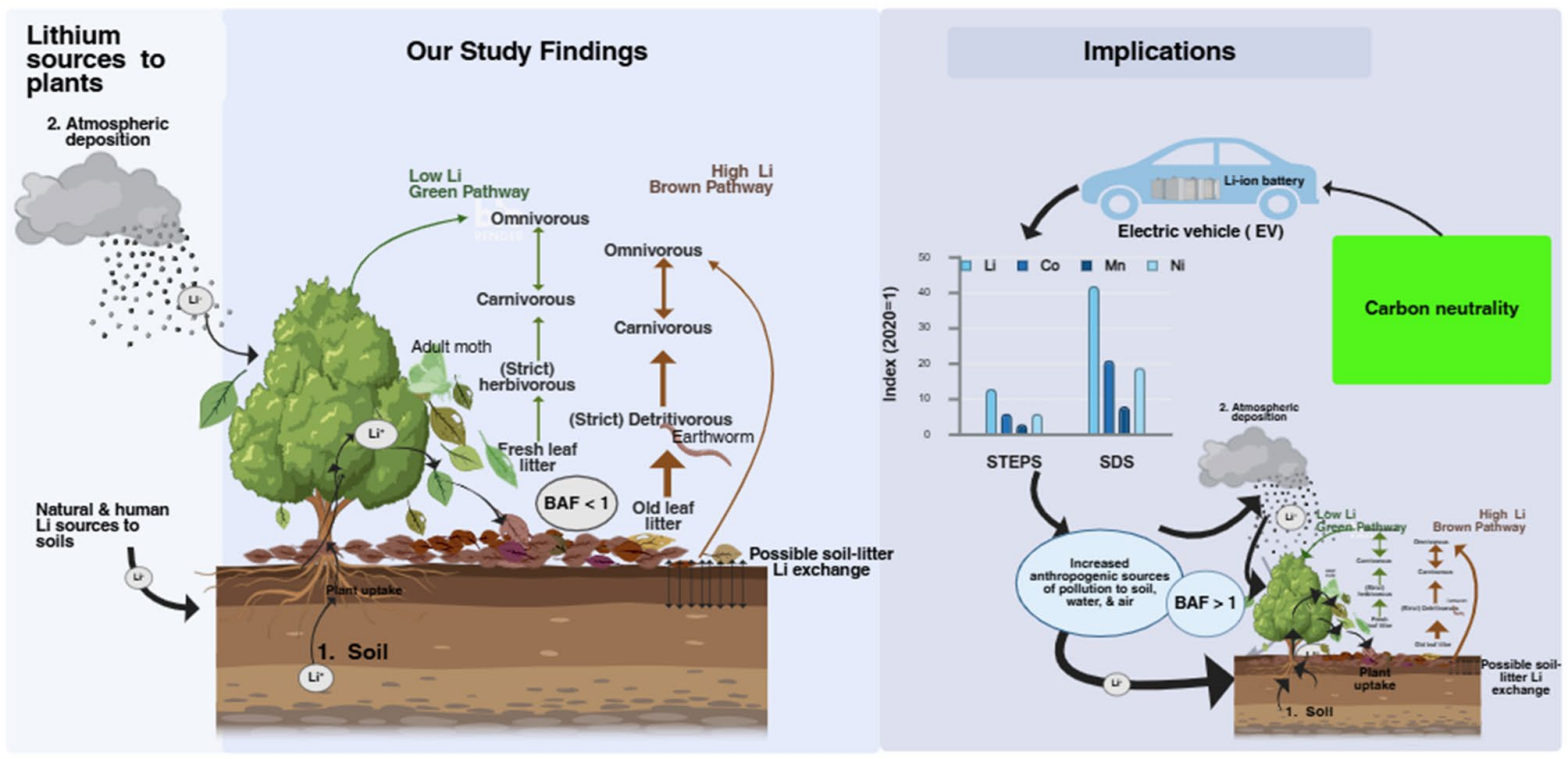
Summary and implications of study findings given the projected Li demand and production of the International Energy Agency (IEA)’s Stated Policies Scenario (STEPS) and Sustainable Development Scenario (SDS). [Bar graph shows the 2040 projected global rise in demand for Li vs. other few selected elements used in clean energy technologies such as electric vehicles (EVs) based on 2020 demand levels (Data source from ref 4).]. The overall figure was created in BioRender. Muisa, N. (2025) https://BioRender.com/9ti9w6y.

Our study hypothesized two distinct Li entry pathways into forest floor food chains: the low Li (“green”: via herbivores) and high Li (“brown”: via detritivores) level pathways, which are based on fresh leaf litter and old leaf litter, respectively (Fig. 6). Given that Li demand and use of Li is set to quadruple while regulation and recycling lag, soil contamination is imminent. An experimental study showed that soil Li contamination increased BAFs of plants ~ 20 times higher than geogenic Li with BAF < 1.2^20^. Consequently, Fig. 6 implies that increased bioavailability and plant uptake and soil contamination would elevate Li transfer to food chains posing a threat to ecosystem health and integrity. Targeted studies on the mechanisms and pathways of Li uptake and elimination, incorporating controlled experiments to elucidate species-specific traits, including the potential role of detritivorous invertebrates in soil Li cycling deserves more attention. Detritivores show potential to act as sink and future reserves of Li in soils warranting future studies in contaminated environments.

## Materials and methods

### Study sites and climatic zones

This study was conducted across four temperate forest sites in the United States (*US*) and two subtropical forest sites in Hong Kong, China (*HK*). Site selection was guided by the need for food web compositions from terrestrial ecosystems of minimal anthropogenic disturbance and similar dominant vegetation (i.e., forest vegetation type). Temperate US forest reserves comprised of Angelo (California); University of Michigan Biological Station (UMBS, Michigan); Coweeta (North Carolina); and Hubbard Brook (New Hampshire) (SI Table S1-S4). Subtropical forests from HK consisted of: Shing Mun Fung Shui Wood (woodland forest) and Tai Po Kau (mixed forest) Nature Reserves (SI Table S5 and S6), which represent typical subtropical forest vegetation cover types found in Hong Kong.

### Sample types and sample collection

We sampled the HK study sites in 2022 whilst the samples from the US forests were collected between 2011 and 2015 as part of a prior published mercury study by Tsui et al.^58^ Samples were collected in the summer season of each forest. In each forest, 2–5 sampling sub-sites were strategically chosen and located at a minimum of 27 m from freshwater bodies, with most beyond 100 m, to avoid aquatic-derived dietary sources^60^^,61^.

In each site, we collected composite samples of the 0–10 cm soil (*n* = 2–3), fresh (O_i_) leaf litter (*n* = 4–5), and invertebrates (*n* = 183), over an average of 4-day trips, in polyethylene ziplock bags using non-powdered, polyvinyl gloves (SI Table S1-S6). We collected soil samples using a stainless-steel hand shovel. Leaf litter was collected using litter traps placed near the sampling sites for invertebrates and soil. We caught insects and non-insects (hereafter named invertebrates for brevity) with pitfall traps (day/night), hand collection with tweezers and dip nets (daytime), and light traps (night) (*see* SI Table S10 for sample distribution). We further categorized the invertebrates by their predominant feeding habit (SI Table S7), and they covered the main feeding guilds/habits (herbivorous, carnivorous, omnivorous, detritivorous, coprophagous).

Across the two regions, the most represented groups included Lepidoptera (moths and butterflies), Coleoptera (mainly, beetles), and Orthoptera (grasshoppers and crickets), with 41, 36, and 20 composite samples each, respectively (SI Table S10). Although the samples were collected during summer in both temperate and subtropical regions, there is differential representation of invertebrates in the studied forests. While our study did not seek to make a one-to-one comparison of the forests but to provide wider coverage instead, we regarded each forest as distinct, providing a trophic transfer pattern unique to its own setting. As such, we reckoned that what is of paramount importance for a trophic transfer study is the broad representation of invertebrate samples across key trophic levels for each forest considered in this study^62^. Besides, the inherent differences within and across forests and climatic regions, including invertebrate species traits (e.g., body size, speed of movement) and niche (e.g., inaccessible habitats, foraging activity), all come into play to influence invertebrate sample distribution and how many of each species can be obtained^29^, making it practically not feasible to ensure same sample invertebrate species numbers.

### Sample processing and analysis for stable isotopes of C and N, and total Li

We processed all samples using standardized protocol adopted from Tsui et al.^58^. Briefly, leaf litter, soils and invertebrate samples were transported to the laboratory, freeze-dried, ground using pestle and mortar or a grinder, and stored in pre-cleaned polypropylene centrifuge tubes (Falcon, Corning) or glass vials with PTFE-lined septa (Thermo Scientific).

We analyzed all biota samples for total carbon, total nitrogen, stable isotopes of carbon (δ^13^C) and nitrogen (δ^15^N) using the gas isotope-ratio mass spectrometer coupled in continuous flow to an elemental analyzer (EA-IRMS) method at both the Stable Isotope Laboratory (SIL), University of Hong Kong, HK and the Colorado Plateau Stable Isotope Laboratory (CPSIL) at Northern Arizona University (Flagstaff, AZ, USA). At CPSIL, repeated analyses of SRM NIST-1547 peach leaves (*n* = 78) produced analytical precision of 0.018‰ for δ¹³C and 0.017‰ for δ¹⁵N (2 SE). At the Stable Isotope Laboratory (SIL), two certified reference materials (CRMs) – USGS-40 (low δ¹⁵N glutamic acid) and USGS-41a (high δ¹⁵N glutamic acid) – were analyzed in triplicate at the start and end of each batch. The mean ± standard deviation (SD) of measured values across all runs (*n* = 12) were δ¹⁵*N* = -2.8 ± 0.06‰ and 48.9 ± 0.18‰, and δ¹³C = -31.8 ± 0.17‰ and 30.3 ± 0.20‰, for USGS-40 and USGS-41a, respectively. Acetanilide (iACET) was used as a secondary analytical standard, with measured values of δ¹⁵*N* = 1.3 ± 0.11‰ and δ¹³C = − 29.6 ± 0.06‰ (*n* = 20). We digested biota (~0.05 g, by a mixture of H_2_O_2_ and HNO_3_ in 1:4 (v:v)) and soil (~0.2 g by a mixture of HCl and HNO_3_ in 1:4 (v:v)) samples for Li analysis alongside reagent blanks and CRMs, with prior cold digestion for both. We employed Agilent 7900 Quadruple-Inductively Coupled Plasma-Mass Spectroscopy (Q-ICP-MS) for all total Li (as Li-7 isotope) analyses. Li-7 isotope is the most abundant (~ 95%) of the stable Li isotopes^26^ and absent in the internal standard. The CRMs used to check for analytical accuracy and Li recovery estimation were: plant: GBW 10,020 (GSB-11) citrus leaf – Institute of Geophysical and Geochemical Exploration, China; animal tissue: DORM-5 fish protein (National Research Council Canada); soil: GBW07405 (GSS-5) yellow-red soil (National Center for Standard Materials, China). The mean CRM recoveries were: [GBW10020: 85.57 ± 4.68 %, *n* = 20; DORM-5: 98.23 ± 9.44 %, *n* = 24; GBW07405 (GSS-5): 94.04 ± 3.46 %, *n* = 4]. Overall, two samples (*n* = 2) were below the detection limit (3.10 ± 2.45 ng/g dry weight) and were excluded from further analyses.

### Quality assurance protocols during sample analysis

To minimize cross-contamination during grinding of biota samples, we cleaned the equipment between samples using deionized water and isopropyl alcohol. The Teflon digestion vessels used during biota sample digestion for Li analysis were pre-washed with 20% HNO₃ and 1% HCl for each digestion run, over two consecutive days. We used EasyPrep™ protocol for post-run cleaning for soil sample iPrep digestion vessels, which included a program consisting of 25-min ramp, 30-min hold at 200 ^o^C, with prior cold digestion for 20 minutes uncapped in the fume hood. It should be noted that we used iPrep™ high-performance microwave vessels (CEM Corporation, USA), on a standard MARS Express microwave system equipped with iWave™ temperature control and a modified turntable to support iPrep™ vessel operation. A reagent blank–CRM–reagent blank analysis protocol was employed after every 10-sample run. Sample digests were filtered with 0.45-µm glass fiber filter and diluted 30 × with Milli-Q purified water before ICP-MS analysis. We performed external standard calibration curves with a working standard prepared from a 1,000 ppm multi-element ICP-MS calibration standard solution (Thermo Fisher Scientific). To correct for instrumental drift and memory effects, we used a 100 ppm multi-element ICP-MS internal standard mix (Thermo Fisher Scientific) at 10 ppb and 50 ppb for biota and soil samples, respectively.

Our results were corrected using beryllium, Be-9. To further assess instrument drift, five (5) random samples were pre-selected and reanalyzed at the end of each batch. Results of Shapiro-Wilk test for normality and Wilcoxon signed-rank test indicated that the repeated measurements significantly deviated from the original runs although the effect was moderate (*p* = 0.02186, *r* = 0.4118). This highlighted the importance of incorporating the internal standard to correct for instrument drift and memory effects.

### Statistical analysis and data presentation

We used Shapiro-Wilk test and Levene’s test to check for normality and homogeneity of variances, respectively. Log transformation normalized the data variability among all groupings. Consequently, the study employed Kruskal-Wallis non-parametric tests with Dunn’s post-hoc test^63^ and Benjamini-Hochberg correction to assess significant differences for two or more groups. Wilcoxon signed rank test with continuity correction was employed for pairwise comparisons between plant litter and invertebrates. The relationships between Li and other quantitative parameters were analyzed using Spearman’s correlation analyses. We used α = 0.05 as the level of significance for all statistical analyses. Therefore, we use median concentrations to report our results. Statistical analyses and data visualization were done using R 4.4.0 (Core Team, 2025).

We further analyzed the data using multiple correspondence analysis (MCA) on the whole dataset (SI Table S1-S6). We used location (forest), feeding habit, and invertebrate order as categorical qualitative variables and log_10_-transformed Li as the supplementary quantitative variable. We presented our MCA biplot results grouped by locations (Fig. 2). Although, MCA does not show the contribution of a factor to the pattern depicted, it is useful to reduce complex datasets (e.g., SI Table S1-S6) into a visual grouping of the coordinates of qualitative variables arranged in a 2-dimensional space by their similarity to each other.

We used stable nitrogen isotope (δ^15^N) data to calculate the trophic levels (TL) of each invertebrate using Eq. 1^37,62^:

1 $$\:T{L}_{\mathrm{invertebrate\:}}=1+\frac{\left({\delta\:}^{15}{\hspace{0.25em}\mathrm{N}}_{\mathrm{invertebrate\:}}-{\delta\:}^{15}{\hspace{0.25em}\mathrm{N}}_{\mathrm{fresh\:litter\:}}\right)}{3.4}$$

where 1 represents the TL of the baseline plant litter, 3.4‰ is the enrichment factor commonly used for natural food web studies^37^. The trophic pattern of Li is obtained from the slope (b) of the linear regression model of log[Li] against δ^15^N using Eq. 2):

2 $$\:{\mathrm{l}\mathrm{o}\mathrm{g}}_{10}\left[\mathrm{L}\mathrm{i}\right]=\mathrm{a}+\mathrm{b}\times\:{\delta\:}^{15}\hspace{0.25em}\mathrm{N}$$

where (a) is the intercept or the log[Li] level of the basal resource; (b) is the slope. We calculated the trophic magnification factor (TMF) using the slope (b) from Eq. (3a) in Eq. (3b).

3a $$\log_{10}[Li] = a+b \times TL $$

3b $$TMF = 10^{b}$$

## Supplementary Information

Supplementary Material 1.

## Data Availability

All data generated or analyzed during this study are included in this published article and its supplementary information (SI) file.
