# Supplementary material for: Biodiminution of lithium in forest floor food webs
Source: Sci Rep. 2026 Apr 3;16:15907. doi: 10.1038/s41598-026-46717-1 (PMC13194703; doi:10.1038/s41598-026-46717-1)
Supplement: Supplementary file 1 — Supplementary Material 1. [file 41598_2026_46717_MOESM1_ESM.docx]

*Supplemental Information (SI)*

**Biodiminution of lithium in terrestrial food webs**

Norah Muisa ^a^, Matthew Long-Hei Cheng ^a,b^, Martin Tsz-Ki Tsui ^a,b,c,*^

*^a^ School of Life Sciences, The Chinese University of Hong Kong, Shatin, N.T., Hong Kong SAR, China*

*^b^ Department of Earth and Environmental Sciences, The Chinese University of Hong Kong, Shatin, N.T., Hong Kong SAR, China*

*^c^ State Key Laboratory of Marine Environmental Health, City University of Hong Kong, Kowloon Tong, Kowloon, Hong Kong SAR, China*

* Corresponding author. **M.T.-K. Tsui**; E-mail: [mtktsui@cuhk.edu.hk](mailto:mtktsui@cuhk.edu.hk)

# Table S1 The sample information and data including forest type, type/order, family, common name, number of samples analyzed (n), carbon to nitrogen (C:N) ratio, stable C and N isotope ratios, estimated trophic level (TL), total Li concentration (ng/g, dry weight basis), and log_10_-transformed Li concentration of different sample types and invertebrate samples collected from forests within the University of Michigan Biological Station (UMBS) (Michigan, USA).

| Forest type | Type/Order | Family | Common name | n | C:N | δ^13^C  (‰) | δ^15^N  (‰) | TL | Total Li  (ng/g) | Log[Li]  (ng/g) |
| --- | --- | --- | --- | --- | --- | --- | --- | --- | --- | --- |
| Coniferous | Soil |  |  |  |  |  |  |  | 6,765.63 | 3.83 |
| Coniferous | Soil |  |  |  |  |  |  |  | 7,541.42 | 3.88 |
| Coniferous | Soil |  |  |  |  |  |  |  | 4,068.31 | 3.61 |
| Coniferous | Leaf litter |  |  |  | 78.3 | -28.8 | -3.1 | 1.01 | 148.53 | 2.17 |
| Coniferous | Leaf litter |  |  |  | 68.7 | -28.5 | -5.3 | 0.37 | 101.65 | 2.01 |
| Deciduous | Leaf litter |  |  |  | 31.9 | -31.3 | -2.4 | 1.21 | 18.55 | 1.27 |
| Deciduous | Leaf litter |  |  |  | 40.4 | -29.4 | -1.9 | 1.34 | 560.01 | 2.75 |
| Deciduous | Leaf litter |  |  |  | 45.2 | -28.7 | -2.9 | 1.07 | 252.87 | 2.40 |
| Coniferous | Lepidoptera | not specified | Moth (mixed) | ~100 | 6.2 | -28.7 | 1.7 | 2.42 | 15.67 | 1.19 |
| Coniferous | Lepidoptera | Geometridae | Geometrid moth | ~100 | 5.7 | -27.7 | 0.5 | 2.05 | 45.91 | 1.66 |
| Coniferous | Lepidoptera | L/N/L | Moth (mixed) | ~100 | 7.3 | -29.5 | 1.1 | 2.24 | 19.89 | 1.30 |
| Deciduous | Lepidoptera | L/N/L | Moth (mixed) | ~100 | 6.0 | -27.3 | 3.9 | 3.07 | 14.19 | 1.15 |
| Deciduous | Lepidoptera | L/N/L | Moth (mixed) | 63 | 7.3 | -27.5 | 2.8 | 2.75 | 42.96 | 1.63 |
| Deciduous | Lepidoptera | Sphingidae | Sphinx moth | 8 | 8.4 | -31.5 | 4.4 | 3.22 | 38.66 | 1.59 |
| Coniferous | Orthoptera | not specified | Grasshopper | 7 | 4.7 | -28.0 | -3.2 | 0.97 | 17.16 | 1.23 |
| Deciduous | Stylommatophora | Arionidae | Land snail |  | 7.2 | -21.4 | 0.8 | 2.14 | 79.52 | 1.90 |
| Coniferous | Stylommatophora | Arionidae | Roundback slugs | 11 | 4.9 | -24.2 | -0.6 | 1.74 | 391.81 | 2.59 |
| Coniferous | Stylommatophora | Arionidae | Roundback slugs | 8 | 6.8 | -23.8 | -0.1 | 1.89 | 90.19 | 1.96 |
| Deciduous | Stylommatophora | Arionidae | Roundback slugs | 21 | 5.4 | -23.7 | 2.0 | 2.51 | 38.06 | 1.58 |
| Deciduous | Stylommatophora | Arionidae | Roundback slugs | 10 | 6.3 | -24.1 | 2.0 | 2.50 | 87.86 | 1.94 |
| Deciduous | Orthoptera | Gryllidae | Cricket | 30 | 4.3 | -23.7 | 4.5 | 3.23 | 55.25 | 1.74 |
| Coniferous | Orthoptera | Gryllidae | Cricket | 4 | 5.3 | -25.8 | 0.9 | 2.17 | 24.11 | 1.38 |
| Deciduous | Orthoptera | Gryllidae | Cricket | 8 | 4.9 | -24.7 | 3.3 | 2.87 | 65.47 | 1.82 |
| Deciduous | Coleoptera | Cucujidae | Flat bark beetle | 3 | 9.7 | -23.9 | 3.4 | 2.57 | 27.16 | 1.43 |
| Coniferous | Coleoptera | Scarabaeidae | May beetle | 60 | 5.3 | -26.2 | 2.2 | 4.67 | 25.73 | 1.41 |
| Coniferous | Coleoptera | Carabidae | Ground beetle | 34 | 6.8 | -26.0 | 1.9 | 3.30 | 25.57 | 1.41 |
| Deciduous | Coleoptera | Carabidae | Carrion beetle | 9 | 7.1 | -26.1 | 9.4 | 2.48 | 45.37 | 1.66 |
| Deciduous | Coleoptera | Carabidae | Ground beetle | 101 | 5.5 | -25.0 | 4.7 | 3.05 | 36.21 | 1.56 |
| Coniferous | Coleoptera | Carabidae | Ground beetle | 20 | 5.8 | -26.2 | 3.9 | 3.47 | 27.88 | 1.45 |
| Deciduous | Coleoptera | Carabidae | Ground beetle | ~50 | 4.5 | -24.8 | 5.3 | 4.01 | 30.19 | 1.48 |
| Deciduous | Coleoptera | Carabidae | Beetle larvae | 87 | 6.6 | -23.0 | 1.2 | 3.5 | 71.45 | 1.85 |
| Coniferous | Diptera | Asilidae | Robber flies | 5 | 4.7 | -25.3 | 7.1 | 4.0 | 30.19 | 1.48 |
| Deciduous | Arachnida | Opiliones | Harvestman | 20 | 4.8 | -26.5 | 7.2 | 4.04 | 25.16 | 1.40 |
| Deciduous | Arachnida | Opiliones | Harvestman | 78 | 5.1 | -27.4 | 6.1 | 3.72 | 163.85 | 2.21 |
| Coniferous | Arachnida | Opiliones | Harvestman | 5 | 4.9 | -27.2 | 1.9 | 2.47 | 223.39 | 2.35 |
| Coniferous | Arachnida | Araneae | Mixed spider | 67 | 4.6 | -25.2 | 3.7 | 3.00 | 124.80 | 2.10 |
| Coniferous | Arachnida | Araneae | Mixed Spider | 25 | 4.7 | -25.5 | 4.1 | 3.12 | 13.71 | 1.14 |
| Deciduous | Arachnida | Araneae | Mixed spider | 26 | 4.5 | -25.9 | 6.5 | 3.82 | 612.31 | 2.79 |
| Deciduous | Spirobolida | Spirobolidae | Millipede |  | 6.2 | -23.6 | -2.2 | 1.26 | 1,059.69 | 3.03 |
| Coniferous | Spirobolida | Spirobolidae | American giant millipede | 18 | 6.0 | -24.1 | -3.4 | 0.92 | 110.78 | 2.04 |
| Deciduous | Spirobolida | Spirobolidae | American giant millipede | 10 | 5.7 | -22.6 | 1.2 | 2.28 | 143.14 | 2.16 |
| Deciduous | Isopoda | Oniscidea | Woodlice | 42 | 4.8 | -22.2 | -0.5 | 1.77 | 186.80 | 2.27 |
| Deciduous | Opisthopora | Lumbricidae | Earthworm |  | 3.2 | -23.5 | 0.6 | 2.09 | 371.43 | 2.57 |
| Deciduous | Opisthopora | Lumbricidae | Earthworm | 10 | 5.7 | -24.3 | 1.4 | 2.32 | 852.14 | 2.93 |

Note: L/N/L= *Lasiocampidae/Noctuidae/Lymantriinae*.

# Table S2 The sample information and data including forest type, type/order, family, common name, number of samples analyzed (n), carbon to nitrogen (C:N) ratio, stable C and N isotope ratios, estimated trophic level (TL), total Li concentration (ng/g, dry weight basis), and log_10_-transformed Li concentration of different sample types and invertebrate samples collected from forests within Angelo Coast Range Reserve (California, USA).

| Forest type | Type/Order | Family | Common name | n | C:N | δ^13^C  (‰) | δ^15^N  (‰) | TL | Total Li  (ng/g) | Log[Li]  (ng/g) |
| --- | --- | --- | --- | --- | --- | --- | --- | --- | --- | --- |
| Mixed forest | Soil |  |  |  |  |  |  |  | 41,799.63 | 4.62 |
| Mixed forest | Soil |  |  |  |  |  |  |  | 39,783.01 | 4.60 |
| Mixed forest | Leaf litter |  |  |  | 198.1 | -28.7 | -3.3 | 0.2 | nd | nd |
| Mixed forest | Leaf litter |  |  |  | 83.4 | -28.8 | -1.9 | 0.7 | nd | nd |
| Mixed forest | Leaf litter |  |  |  | 98.8 | -27.8 | -1.6 | 0.7 | nd | nd |
| Mixed forest | Leaf litter |  |  |  | 49.2 | -29.5 | 3.9 | 2.4 | 4,496.72 | 3.65 |
| Mixed forest | Lepidoptera | Mixed | Stout moths | 140 | 6.4 | -27.9 | 1.7 | 1.7 | 31.44 | 1.50 |
| Mixed forest | Lepidoptera | Mixed | Moths | 49 | 5.7 | -26.0 | 2.1 | 1.8 | 45.82 | 1.66 |
| Mixed forest | Lepidoptera | Geometridae | Geometrid moths | 42 | 5.5 | -26.0 | 3.8 | 2.3 | 73.55 | 1.87 |
| Mixed forest | Orthoptera | Calaifera | Grasshopper | 58 | 9.3 | -27.5 | -0.6 | 1.0 | 356.08 | 2.55 |
| Mixed forest | Stylommatophora | Arionidae | Roundback slug | 8 | 6.8 | -23.9 | 1.0 | 1.5 | 45.06 | 1.65 |
| Mixed forest | Orthoptera | Gryllidae | Field cricket | 11 | 5.2 | -24.3 | -0.7 | 1.0 | 392.59 | 2.59 |
| Mixed forest | Orthoptera | Stenopelmatidae | Jerusalem cricket | 4 | 5.1 | -24.1 | 4.6 | 2.6 | 2,191.06 | 3.34 |
| Mixed forest | Arachnida | Opiliones | Harvestman | 32 | 5.4 | -25.9 | 2.8 | 2.0 | 7,301.92 | 3.86 |
| Mixed forest | Coleoptera | Phengodidae | Glowworm beetle larvae | 1 | 9.7 | -24.8 | 8.2 | 4.10 | 134.9967 | 1.73 |
| Mixed forest | Coleoptera | Carabidae | Carabid beetle | 76 | 5.7 | -24.3 | 2.5 | 1.94 | 287.16 | 2.46 |
| Mixed forest | Arachnida | Araneae | Spider (mixed) | 20 | 5.6 | -25.4 | 3.0 | 2.11 | 1.24 | 0.09 |
| Mixed forest | Arachnida | Araneae | Flimy dome spider | 5 |  | -25.1 | 2.6 | 1.99 | 5.95 | 0.77 |
| Mixed forest | Arachnida | Araneae | Orb weaver spider | 110 | 5.6 | -25.6 | 2.5 | 1.95 | 84.47 | 1.93 |
| Mixed forest | Arachnida | Araneae | Spider (mixed) | 95 | 5.2 | -25.0 | 5.4 | 2.82 | 7,393.56 | 3.87 |
| Mixed forest | Arachnida | Araneae | Spider (mixed) | 84 | 5.0 | -24.0 | 5.3 | 2.77 | 6,842.79 | 3.84 |
| Mixed forest | Hemiptera | Largidae | Bordered plant bug | 7 | 6.6 | -25.3 | 0.9 | 1.48 | 110.12 | 2.04 |
| Mixed forest | Hemiptera | Largidae | Bordered plant bug | 35 | 7.1 | -24.3 | 0.7 | 1.42 | 1,550.91 | 3.19 |
| Mixed forest | Scolopendromorpha | Scolocryptoidae | Bark centipede | 9 | 5.5 | -24.6 | 4.3 | 2.47 | 143.15 | 2.16 |
| Mixed forest | Arachnida | Scorpiones | Scorpion (large) | 10 | 5.3 | -24.8 | 5.5 | 2.84 | 76.70 | 1.88 |
| Mixed forest | Arachnida | Scorpiones | Scorpion (small) | 7 | 4.8 | -24.7 | 6.8 | 3.20 | 127.31 | 2.10 |
| Mixed forest | Arachnida | Scorpiones | Scorpion (large) | 2 | 5.8 | -24.5 | 6.2 | 3.02 | 33.07 | 1.52 |
| Mixed forest | Arachnida | Scorpiones | Forest scorpion (small) | 3 | 6.3 | -24.4 | 6.2 | 3.03 | 24.07 | 1.38 |
| Mixed forest | Arachnida | Scorpiones | Scorpion (large) | 2 | 6.1 | -25.4 | 5.6 | 2.87 | 49.05 | 1.69 |
| Mixed forest | Hemiptera | Cicadidae | Cicada | 1 | 4.5 | -23.7 | -0.5 | 1.06 | 4.25 | 0.63 |
| Mixed forest | Spirobolida | Spirobolidae | American giant millipede | 23 | 8.3 | -24.8 | 1.1 | 1.54 | 843.01 | 2.93 |
| Mixed forest | Polydesmida | Polydesmida | Flat millipede | 17 | 7.0 | -23.4 | 1.7 | 1.71 | 1,100.01 | 3.04 |
| Mixed forest | Spirobolida | Spirobolidae | American giant millipede | 13 | 8.2 | -23.8 | 1.8 | 1.7 | 717.16 | 2.86 |

Note: L/N/L= *Lasiocampidae/Noctuidae/Lymantriinae*.

# Table S3 The sample information and data including forest type, type/order, family, common name, number of samples analyzed (n), carbon to nitrogen (C:N) ratio, stable C and N isotope ratios, estimated trophic level (TL), total Li concentration (ng/g, dry weight basis), and log_10_-transformed Li concentration of different sample types and invertebrate samples collected from forests within Coweeta LTER (North Carolina, USA).

| Forest type | Type/Order | Family | Common name | n | C:N | δ^13^C  (‰) | δ^15^N  (‰) | TL | Total Li  (ng/g) | Log[Li]  (ng/g) |
| --- | --- | --- | --- | --- | --- | --- | --- | --- | --- | --- |
| Deciduous |  | Soil |  |  |  |  |  |  | 18,658.42 | 4.27 |
| Deciduous |  | Soil |  |  |  |  |  |  | 9,387.49 | 3.97 |
| Mixed forest |  | Leaf litter |  |  | 44.6 | -29.5 | -1.7 | 1.30 | 178.94 | 2.25 |
| Coniferous |  | Leaf litter |  |  | 95.3 | -29.4 | -2.8 | 0.99 | 121.44 | 2.08 |
| Coniferous |  | Leaf litter |  |  | 48.2 | -29.6 | -3.2 | 0.87 | 199.05 | 2.30 |
| Coniferous |  | Leaf litter |  |  | 38.1 | -28.4 | -3.4 | 0.83 | 207.52 | 2.32 |
| Coniferous | Lepidoptera | Geometridae | Moth larvae | ~200 | 6.3 | -29.9 | 1.4 | 2.22 | 364.23 | 2.56 |
| Deciduous | Lepidoptera | L/N/L | Mixed moths | 100 | 5.7 | -30.3 | 1.2 | 2.17 | 9.62 | 0.98 |
| Deciduous | Lepidoptera | Geometridae | Geometrid moths | ~100 | 5.0 | -28.9 | 0.1 | 1.84 | 68.72 | 1.84 |
| Deciduous | Lepidoptera | Bombycidae | Silkworm moths | 4 | 7.5 | -30.2 | 3.7 | 2.92 | 2.39 | 0.38 |
| Mixed forest | Lepidoptera | L/N/L | Mixed moths | ~200 | 6.7 | -30.3 | 0.9 | 2.08 | 10.84 | 1.04 |
| Mixed forest | Lepidoptera | Geometridae | Geometrid moths | ~100 | 5.2 | -28.7 | 0.6 | 1.98 | 5.90 | 0.77 |
| Coniferous | Lepidoptera | Sphingidae | Sphinx moth | 7 | 9.0 | -32.8 | 2.8 | 2.64 | 29.47 | 1.47 |
| Deciduous | Lepidoptera | Geometridae | Geometrid moths | ~100 | 4.9 | -28.2 | 0.4 | 1.93 | 27.82 | 1.44 |
| Mixed forest | Lepidoptera | not specified | Butterfly | 22 | 3.0 | -30.4 | 2.5 | 2.55 | 71.01 | 1.85 |
| Coniferous | Stylommatophora | Arionidae | Dusky slug | 11 | 6.0 | -24.6 | 0.3 | 1.91 | 15.60 | 1.19 |
| Deciduous | Stylommatophora | Arionidae | Dusky slug | 6 | 7.7 | -24.2 | 3.2 | 2.75 | 26.63 | 1.43 |
| Coniferous | Stylommatophora | Arionidae | Land snail | 1 | 7.0 | -24.6 | 1.4 | 2.21 | 271.44 | 2.43 |
| Coniferous | Stylommatophora | Arionidae | Land snail | 32 | 6.3 | -22.6 | 1.5 | 2.26 | 46.34 | 1.67 |
| Deciduous | Stylommatophora | Arionidae | Land snail | 17 | 7.0 | -21.0 | 3.1 | 2.73 | 28.23 | 1.45 |
| Coniferous | Orthoptera | Rhaphidophoridae | Camal cricket | 5 | 4.6 | -25.6 | 4.2 | 3.06 | 297.15 | 2.47 |
| Coniferous | Orthoptera | Gryllidae | Field cricket | 10 | 4.8 | -25.7 | 2.6 | 2.58 | 46.88 | 1.67 |
| Mixed forest | Orthoptera | Gryllidae | Field cricket | 9 | 5.0 | -25.3 | 2.6 | 2.59 | 66.70 | 1.82 |
| Coniferous | Arachnida | Opiliones | Harvestman | 34 | 4.7 | -27.1 | 2.6 | 2.59 | 120.01 | 2.08 |
| Mixed forest | Coleoptera | Elateridae | Click beetle | 4 | 4.6 | -25.1 | 2.7 | 2.62 | 166.38 | 2.22 |
| Coniferous | Coleoptera | Elateridae | Click beetle | 6 | 6.2 | -24.0 | 2.0 | 2.40 | 32.87 | 1.52 |
| Deciduous | Coleoptera | Carabidae | Ground beetle | 10 | 5.5 | -27.1 | 3.0 | 2.69 | 123.80 | 2.09 |
| Deciduous | Coleoptera | Carabidae | Ground beetle | 20 | 6.0 | -25.9 | 2.6 | 2.57 | 58.93 | 1.77 |
| Coniferous | Coleoptera | Carabidae | Ground beetle | ~20 | 5.4 | -25.7 | 3.0 | 2.70 | 62.01 | 1.79 |
| Mixed forest | Coleoptera | Carabidae | Ground beetle | 2 | 4.6 | -25.9 | 3.6 | 2.89 | 211.33 | 2.32 |
| Coniferous | Coleoptera | Carabidae | Ground beetle | 35 | 5.1 | -25.8 | 3.2 | 2.77 | 236.69 | 2.37 |
| Mixed forest | Coleoptera | Silphidae | Carrion beetle | 6 | 7.6 | -25.9 | 5.4 | 3.39 | 59.30 | 1.77 |
| Deciduous | Coleoptera | Silphidae | Carrion beetle | 3 | 4.7 | -23.7 | 7.1 | 3.91 | 64.43 | 1.81 |
| Deciduous | Arachnida | Lycosidae | Wolf spiders | 3 | 4.1 | -25.6 | 3.5 | 2.84 | 124.48 | 2.10 |
| Deciduous | Arachnida | Araneae | Ground spider | 10 | 4.6 | -25.4 | 3.7 | 2.91 | 305.89 | 2.49 |
| Coniferous | Arachnida | Araneae | Ground spider | 20 | 4.7 | -25.7 | 2.9 | 2.66 | 57.98 | 1.76 |
| Deciduous | Scolopendromorpha | Lithobiidae | Stone centipede | 11 | 5.3 | -25.5 | 2.8 | 2.63 | 293.63 | 2.47 |
| Coniferous | Scolopendromorpha | Lithobiidae | Stone centipede | ~5 | 4.6 | -24.3 | 2.9 | 2.65 | 49.08 | 1.69 |
| Coniferous | Blattodea | Ectobiidae | Wood roaches | 5 | 5.0 | -24.4 | -0.9 | 1.56 | 71.73 | 1.86 |
| Deciduous | Blattodea | Cryptocercidae | Woodroaches | 2 | 4.7 | -24.1 | -2.3 | 1.15 | 140.87 | 2.15 |
| Deciduous | Spirobolida | Spirobolidae | American giant millipede | 7 | 6.7 | -26.1 | -3.2 | 0.87 | 87.87 | 1.94 |
| Deciduous | Polydesmida | Xystodesmidae | Flat millipedes | 7 | 6.3 | -22.4 | 0.6 | 1.99 | 82.72 | 1.92 |
| Deciduous | Spirobolida | Spirobolidae | American giant millipede | 10 | 6.9 | -26.2 | -2.7 | 1.03 | 171.83 | 2.24 |
| Coniferous | Polydesmida | Xystodesmidae | Flat millipedes | 5 | 5.3 | -24.2 | 0.2 | 1.88 | 1,875.89 | 3.27 |
| Coniferous | Spirobolida | Spirobolidae | American giant millipede | 10 | 6.8 | -25.5 | -1.6 | 1.34 | 259.37 | 2.41 |
| Deciduous | Spirobolida | Spirobolidae | American giant millipede | ~15 | 6.7 | -24.0 | -0.2 | 1.76 | 405.40 | 2.61 |
| Mixed, secondary | Spirobolida | Spirobolidae | American giant millipede | ~15 | 6.0 | -21.9 | 1.4 | 2.23 | 236.92 | 2.37 |
| Mixed, secondary | Opisthopora | Lumbricidae | Earthworm | 20 | 4.6 | -24.6 | -1.1 | 1.48 | 117.61 | 2.07 |
| Mixed, secondary | Opisthopora | Lumbicidae | Earthworm | 1 | 7.0 | -25.3 | 2.0 | 2.41 | 7,891.16 | 3.90 |

Note: L/N/L= *Lasiocampidae/Noctuidae/Lymantriinae*.

# Table S4 The sample information and data including forest type, type/order, family, common name, number of samples analyzed (n), carbon to nitrogen (C:N) ratio, stable C and N isotope ratios, estimated trophic level (TL), total Li concentration (ng/g, dry weight basis), and log_10_-transformed Li concentration of different sample types and invertebrate samples collected from forests within Hubbard Brook Experimental Forest (New Hampshire, USA).

| Forest type | Type/Order | Family | Common name | n | C:N | δ^13^C  (‰) | δ^15^N  (‰) | TL | Total Li  (ng/g) | Log[Li]  (ng/g) |
| --- | --- | --- | --- | --- | --- | --- | --- | --- | --- | --- |
| Mixed forest | Soil |  |  |  |  |  |  |  | 3,346.59 | 0.52 |
| Mixed forest | Leaf litter |  |  |  | 36.5 | -30.0 | -2.1 | 1.0 | 295.50 | 2.47 |
| Mixed forest | Leaf litter |  |  |  | 34.3 | -29.5 | -1.7 | 1.1 | 102.60 | 2.01 |
| Mixed forest | Leaf litter |  |  |  | 32.5 | -29.3 | -2.3 | 0.9 | 85.90 | 1.93 |
| Mixed forest | Lepidoptera | Sphingidae | Sphinx moth | 11 | 6.8 | -31.0 | 3.6 | 2.7 | 30.59 | 1.49 |
| Mixed forest | Lepidoptera | Sphingidae | Sphinx moth | 11 | 6.3 | -29.8 | 3.8 | 2.7 | 32.19 | 1.51 |
| Mixed forest | Lepidoptera | Erebidae | Tiger moth | 13 | 7.9 | -34.5 | 3.6 | 2.7 | 19.21 | 1.28 |
| Mixed forest | Lepidoptera | L/N/L | Moth (mixed) | ~100 | 7.3 | -30.5 | 3.6 | 2.7 | 6.39 | 0.81 |
| Mixed forest | Lepidoptera | L/N/L | Moth (mixed) | ~200 | 6.1 | -29.9 | 3.6 | 2.6 | 6.43 | 0.81 |
| Mixed forest | Lepidoptera | Geometridae | Geometrid moth | ~100 | 5.3 | -28.1 | 3.1 | 2.5 | 26.27 | 1.42 |
| Mixed forest | Lepidoptera | Saturniie | Polyphemus | 6 | 5.3 | -29.1 | 3.2 | 2.6 | 223.99 | 2.35 |
| Mixed forest | Stylommatophora | Arionidae | Dusky slug | 8 | 5.7 | -22.6 | 1.8 | 2.1 | 41.45 | 1.62 |
| Mixed forest | Coleoptera | Lycidae | Net wing beetle | 19 | 7.6 | -25.8 | 2.7 | 2.4 | 16.40 | 1.21 |
| Mixed forest | Coleoptera | Pyrochroidae | Fire colored beetle | 10 | 5.6 | -23.8 | 2.5 | 2.3 | 33.57 | 1.53 |
| Mixed forest | Coleoptera | Carabidae | Carabid beetle | 50 | 6.1 | -26.6 | 4.2 | 2.8 | 68.60 | 1.84 |
| Mixed forest | Araneae | Araneae | Ground spider | 31 | 4.7 | -25.0 | 5.9 | 3.3 | 175.40 | 2.24 |

Note: L/N/L= *Lasiocampidae/Noctuidae/Lymantriinae*.

# Table S5 The sample information and data including forest type, type/order, family, common name, number of samples analyzed (n), carbon to nitrogen (C:N) ratio, stable C and N isotope ratios, estimated trophic level (TL), total Li concentration (ng/g, dry weight basis), and log_10_-transformed Li concentration of different sample types and invertebrate samples collected from forests within Tai Po Kau Nature Reserve (Hong Kong, China).

| Forest type | Order | Family | Common name | n | C:N | δ^13^C  (‰) | δ^15^N  (‰) | TL | Total Li  (ng/g) | Log[Li]  (ng/g) |
| --- | --- | --- | --- | --- | --- | --- | --- | --- | --- | --- |
| Mixed | Soil |  |  |  |  |  |  |  | 9,996.92 | 4.00 |
| Mixed | Soil |  |  |  |  |  |  |  | 12,707.13 | 4.10 |
| Mixed | Leaf litter |  | Fresh litter (O_i_) |  | 32.2 | -33.3 | -1.1 | 1.00 | 157.47 | 2.20 |
| Mixed | Leaf litter |  | Old litter (O_e_) |  | 46.1 | -32.8 | -2.1 | 0.72 | 559.31 | 2.75 |
| Mixed | Leaf litter |  | Fresh litter (O_i_) |  | 75.0 | -32.7 | -3.2 | 0.38 | 130.55 | 2.12 |
| Mixed | Leaf litter |  | Old litter (O_e_) |  | 41.3 | -32.5 | -1.5 | 0.88 | 709.99 | 2.85 |
| Mixed | Lepidoptera | Erebidae | Moth | 4 | 4.0 | -29.8 | 3.1 | 2.23 | 80.48 | 1.91 |
| Mixed | Lepidoptera | Erebidae | Moth | 1 | 3.7 | -27.3 | 3.2 | 2.26 | 65.73 | 1.82 |
| Mixed | Lepidoptera | Geometridae | Moth | 16 | 4.4 | -32.2 | 1.8 | 1.85 | 62.52 | 1.80 |
| Mixed | Lepidoptera | not specified | Moth | 60 | 5.6 | -30.5 | 1.3 | 1.70 | 22.87 | 1.36 |
| Mixed | Lepidoptera | Erebidae | Moth | 19 | 5.2 | -30.0 | 3.9 | 2.47 | 96.96 | 1.99 |
| Mixed | Lepidoptera | Yponomeutidae | Moth | 13 | 5.3 | -26.5 | 9.7 | 4.18 | 21.44 | 1.33 |
| Mixed | Phasmida | Phasmatodae | Stick insect | 1 | 5.1 | -30.0 | -0.3 | 1.23 | 193.14 | 2.29 |
| Mixed | Hemiptera | Pentantomidae | Stink bugs | 12 | 5.1 | -28.7 | 0.9 | 1.59 | 31.44 | 1.50 |
| Mixed | Orthoptera | Catantopidae | Short-horned grasshoppers | 1 | 13.2 | -31.4 | 1.5 | 1.76 | 22.69 | 1.36 |
| Mixed | Orthoptera | Catantopidae | Oriental spur-throated grasshopper | 1 | 4.4 | -31.2 | 0.8 | 1.56 | 1,623.13 | 3.21 |
| Mixed | Orthoptera | Catantopidae | Oriental spur-throated grasshopper | 1 | 4.6 | -32.4 | 1.6 | 1.79 | 138.59 | 2.14 |
| Mixed | Orthoptera | Catantopidae | Short-horned grasshoppers | 2 | 4.1 | -20.7 | 3.4 | 2.32 | 69.87 | 1.84 |
| Mixed | Stylommatophora | Ariophantidae | Land snail | 1 | 5.4 | -26.0 | 2.5 | 2.07 | 64.43 | 1.81 |
| Mixed | Orthoptera | Gryllidae | Cricket | 5 | 5.6 | -27.2 | 3.6 | 2.38 | 212.02 | 2.33 |
| Mixed | Scolopendromorpha | Lithobiomorpha | Centipede | 1 | 3.9 | -21.6 | 2.9 | 2.18 | 299.41 | 2.48 |
| Mixed | Scolopendromorpha | Lithobiomorpha | Centipede | 1 | 4.0 | -27.6 | 5.2 | 2.85 | 56.04 | 1.75 |
| Mixed | Arachnida | Ischnuridae | Scorpion | 18 | 4.4 | -27.1 | 5.7 | 3.00 | 240.64 | 2.38 |
| Mixed | Coleoptera | Lampyridae | Firefly | 2 | 6.1 | -25.1 | 7.0 | 3.38 | 108.37 | 2.03 |
| Mixed | Coleoptera | Scarabaeidae | Scarab beetle | 1 | 6.8 | -29.3 | 6.7 | 3.29 | 24.70 | 1.39 |
| Mixed | Coleoptera | Lucanidae | Dung beetle | 1 | 6.0 | -28.9 | 6.4 | 3.20 | 28.53 | 1.46 |
| Mixed | Hymenoptera | Formicidae | Ant | 14 | 4.3 | -27.3 | 2.9 | 2.18 | 488.96 | 2.69 |

# Table S6 The sample information and data including forest type, type/order, family, common name, number of samples analyzed (n), carbon to nitrogen (C:N) ratio, stable C and N isotope ratios, estimated trophic level (TL), total Li concentration (ng/g, dry weight basis), and log_10_-transformed Li concentration of different sample types and invertebrate samples collected from forests within Shing Mun Country Park (Hong Kong, China).

| Forest type | Type/Order | Family | Common name | n | C:N | δ^13^C  (‰) | δ^15^N  (‰) | TL | Total Li  (ng/g) | Log[Li]  (ng/g) |
| --- | --- | --- | --- | --- | --- | --- | --- | --- | --- | --- |
| Woodland | Soil |  |  |  |  |  |  |  | 15,063.23 | 4.18 |
| Woodland | Soil |  |  |  |  |  |  |  | 13,238.33 | 4.12 |
| Woodland | Leaf litter |  | Fresh litter (O_i_) |  | 34.1 | -31.9 | -0.5 | 1.18 | 70.44 | 1.85 |
| Woodland | Leaf litter |  | Fresh litter (O_i_) |  | 38.0 | -31.6 | -0.7 | 1.13 | 236.22 | 2.37 |
| Woodland | Leaf litter |  | Old litter (O_e_) |  | 42.0 | -31.5 | 0.0 | 1.32 | 1,183.88 | 3.07 |
| Woodland | Leaf litter |  | Fresh litter (O_i_) |  | 34.1 | -31.9 | -0.5 | 1.18 | 183.16 | 2.26 |
| Woodland | Leaf litter |  | Old litter (O_e_) |  | 43.4 | -31.3 | -0.8 | 1.09 | 1,304.53 | 3.12 |
| Woodland | Leaf litter |  | Fresh litter (O_i_) |  | 38.0 | -31.6 | -0.7 | 1.13 | 271.58 | 2.43 |
| Woodland | Lepidoptera | Geometridae | Moth larvae | 1 | 4.7 | -30.9 | 3.0 | 2.00 | 123.02 | 1.88 |
| Woodland | Lepidoptera | Erebidae | Moth | 26 | 4.1 | -29.5 | -4.0 | 2.52 | 77.00 | 1.89 |
| Woodland | Lepidoptera | Noctuidae | Moth | 3 | 5.7 | -32.3 | 5.5 | 2.94 | 157.16 | 2.20 |
| Woodland | Lepidoptera | G/E | Moth | 22 | 4.5 | -30.1 | 1.8 | 1.85 | 44.04 | 1.64 |
| Woodland | Lepidoptera | not specified | Moth | 1 | 4.51 | -30.1 | 1.8 | 2.07 | 21.84 | 1.34 |
| Woodland | Lepidoptera | Erebidae | Moth | 3 | 4.3 | -29.5 | 5.6 | 2.97 | 385.61 | 2.59 |
| Woodland | Lepidoptera | Geometridae | Moth | 15 | 4.0 | -31.1 | 2.8 | 2.15 | 62.13 | 1.79 |
| Woodland | Lepidoptera | not specified | Moth | 1 | 4.16 | -29.5 | 0.8 | 1.88 | 91.44 | 1.96 |
| Woodland | Lepidoptera | Nymphalidae | Brush-footed butterfly | 4 | 4.2 | -28.1 | 1.9 | 1.94 | 113.62 | 2.06 |
| Woodland | Lepidoptera | Papilionidae | Swallowtail butterfly | 2 | 4.2 | -30.0 | 2.1 | 2.20 | 183.89 | 2.26 |
| Woodland | Lepidoptera | Nymphalidae | Brush-footed butterfly | 5 | 3.8 | -22.1 | 3.0 | 1.85 | 85.39 | 1.93 |
| Woodland | Orthoptera | not specified | Grasshopper | 1 | 3.8 | -32.4 | 1.8 | 1.29 | 42.62 | 1.63 |
| Woodland | Orthoptera | not specified | Grasshopper | 1 | 4.1 | -32.0 | -0.1 | 2.14 | 245.68 | 2.39 |
| Woodland | Hemiptera | Pentantomidae | Stink bugs | 1 | 3.8 | -29.0 | 2.8 | 1.47 | 131.44 | 2.12 |
| Woodland | Stylommatophora | Ariophantidae | Arboreal/Tree snail | 1 | 5.1 | -27.7 | 0.5 | 1.41 | 198.82 | 2.30 |
| Woodland | Stylommatophora | Ariophantidae | Arboreal/Tree snail | 1 | 7.3 | -26.6 | 0.3 | 1.50 | 138.31 | 2.14 |
| Woodland | Orthoptera | Pseudophyllie | Bush cricket | 1 | 5.0 | -27.9 | 0.6 | 1.91 | 62.37 | 1.79 |
| Woodland | Orthoptera | Gryllidae | Tree cricket | 1 | 3.4 | -28.9 | 2.0 | 1.59 | 49.72 | 1.70 |
| Woodland | Orthoptera | Phaneropteridae | Burrowing cricket | 1 | 4.1 | -28.2 | 0.9 | 1.47 | 67.02 | 1.83 |
| Woodland | Blattodea | Blaberidae | Amphibious litter cockroach | 2 | 3.9 | -28.5 | 0.5 | 1.29 | 73.56 | 1.87 |
| Woodland | Blattodea | Blaberidae | Amphibious litter cockroach | 2 | 3.6 | -26.8 | -0.1 | 1.53 | 120.24 | 2.08 |
| Woodland | Blattodea | Blaberidae | Amphibious litter cockroach | 6 | 3.8 | -27.6 | 0.7 | 1.44 | 3,469.40 | 3.54 |
| Woodland | Blattodea | Blaberidae | Amphibious litter cockroach | 2 | 4.7 | -28.2 | 0.4 | 1.32 | 199.45 | 2.30 |
| Woodland | Blattodea | Blaberidae | Amphibious litter cockroach | 5 | 3.8 | -28.1 | 0.0 | 1.11 | 158.74 | 2.20 |
| Woodland | Blattodea | Blaberidae | Amphibious litter cockroach | 1 | 4.1 | -28.1 | -0.7 | 0.94 | 67.77 | 1.83 |
| Woodland | Blattodea | Blaberidae | Amphibious litter cockroach | 1 | 4.7 | -29.2 | -1.3 | 1.41 | 131.26 | 2.12 |
| Woodland | Blattodea | Blaberidae | Amphibious litter cockroach | 2 | 4.1 | -27.5 | 0.3 | 2.76 | 51.03 | 1.71 |
| Woodland | Coleoptera | Scarabaeidae | Chaffer beetles | 1 | 4.0 | -25.9 | 4.9 | 2.12 | 32.81 | 1.52 |
| Woodland | Coleoptera | Scarabaeidae | Scarab beetle | 5 | 3.9 | -27.5 | 2.7 | 1.85 | 81.28 | 1.91 |
| Woodland | Coleoptera | Scarabaeidae | Scarab beetle | 24 | 4.5 | -29.7 | 1.8 | 3.09 | 66.62 | 1.82 |
| Woodland | Coleoptera | Scarabaeidae | Scarab beetle | 8 | 5.4 | -27.9 | 6.0 | 1.92 | 144.01 | 2.16 |
| Woodland | Coleoptera | Scarabaeidae | Scarab beetle | 1 | 4.3 | -29.0 | 2.0 | 1.73 | 6.05 | 0.78 |
| Woodland | Coleoptera | Lucanidae | Dung beetle | 2 | 6.4 | -29.8 | 1.4 | 2.00 | 130.01 | 2.11 |
| Woodland | Coleoptera | Scarabaeidae | Scarab beetle | 8 | 3.9 | -27.0 | 2.3 | 2.70 | 28.41 | 1.45 |
| Woodland | Coleoptera | Scarabaeidae | Scarab beetle | 3 | 4.0 | -28.3 | 4.7 | 1.69 | 63.66 | 1.80 |
| Woodland | Coleoptera | Scarabaeidae | Scarab beetle | 1 | 3.9 | -28.0 | 1.2 | 2.70 | 56.59 | 1.75 |
| Woodland | Coleoptera | Scarabaeidae | Scarab beetle | 2 | 4.0 | -27.8 | 4.7 | 1.73 | 56.82 | 1.75 |
| Woodland | Coleoptera | Scarabaeidae | Scarab beetle | 21 | 4.4 | -29.4 | 1.4 | 3.41 | 82.21 | 1.91 |
| Woodland | Scolopendromorpha | Lithobiomorpha | Centipede | 1 | 4.5 | -25.9 | 7.1 | 4.12 | 31.32 | 1.50 |
| Woodland | Hymenoptera | Scoliidae | Scoliid wasp | 45 | 4.2 | -25.6 | 9.5 | 1.23 | 42.87 | 1.63 |
| Woodland | Blattodea | Termitidae | Termites | many | 5.7 | -29.9 | -0.3 | 2.00 | 6,580.87 | 3.82 |
| Woodland | Opisthopora | Lumbricidae | Earthworm | 2 | 4.1 | -27.2 | 2.3 | 2.73 | 6,629.43 | 3.82 |
| Woodland | Opisthopora | Lumbricidae | Earthworm | 2 | 3.8 | -26.0 | 4.8 | 1.30 | 1,659.55 | 3.22 |
| Woodland | Opisthopora | Lumbricidae | Earthworm | 2 | 4.2 | -27.6 | -0.1 | 2.20 | 5,066.89 | 3.70 |
| Woodland | Opisthopora | Lumbricidae | Earthworm | 2 | 4.9 | -27.5 | 3.0 | 2.01 | 5012.81 | 3.70 |

# Table S7 Summary statistics [(counts as number of invertebrates, their percentage proportion of total measured invertebrates (%)), the mean, standard deviation (SD), maximum, and minimum of the total Li concentrations - as ng/g on a dry weight basis] of the invertebrates (*n*=178) analyzed and reported in the study across US and HK forests combined, grouped by their feeding habit and common names. [* nd means not determined due to inadequate sample size]

| Feeding habit & Common name | Count | Proportion  (%) | Mean  Li  (ng/g) | SD | Minimum  Li  (ng/g) | Maximum  Li  (ng/g) |
| --- | --- | --- | --- | --- | --- | --- |
| Herbivorous | **58** | **32.6%** | **124.5** | **290.0** | **2.4** | **1,623.1** |
| Bordered plant bug | 2 | 1.1% | 830.5 | 1,018.8 | 110.1 | 1,550.9 |
| Brush-footed butterfly | 2 | 1.1% | 137.7 | 65.4 | 91.4 | 183.9 |
| Butterfly (unspecified) | 1 | 0.6% | 71.0 | nd | 71.0 | 71.0 |
| Chaffer beetles | 1 | 0.6% | 51.0 | nd | 51.0 | 51.0 |
| Click beetle | 2 | 1.1% | 99.6 | 94.4 | 32.9 | 166.4 |
| Dusky slug | 1 | 0.6% | 41.5 | nd | 41.5 | 41.5 |
| Flat bark beetle | 1 | 0.6% | 26.9 | nd | 26.9 | 26.9 |
| Geometrid moth | 11 | 6.2% | 42.0 | 22.5 | 5.9 | 73.5 |
| Grasshopper (unspecified) | 4 | 2.2% | 125.3 | 156.4 | 17.2 | 356.1 |
| May beetle | 1 | 0.6% | 27.2 | nd | 27.2 | 27.2 |
| Moth | 7 | 3.9% | 126.3 | 121.3 | 21.4 | 385.6 |
| Moth (mixed) | 5 | 2.8% | 19.5 | 13.7 | 9.6 | 43.0 |
| Net wing beetle | 1 | 0.6% | 16.4 | nd | 16.4 | 16.4 |
| Oriental spur-throated grasshopper | 2 | 1.1% | 880.9 | 1,049.7 | 138.6 | 1,623.1 |
| Polyphemus | 1 | 0.6% | 224.0 | nd | 224.0 | 224.0 |
| Short-horned grasshopper | 2 | 1.1% | 46.3 | 33.4 | 22.7 | 69.9 |
| Silkworm moths | 1 | 0.6% | 2.4 | nd | 2.4 | 2.4 |
| Sphinx moth | 4 | 2.2% | 32.7 | 4.1 | 29.5 | 38.7 |
| Stick insect | 1 | 0.6% | 193.1 | nd | 193.1 | 193.1 |
| Stink bugs | 2 | 1.1% | 138.6 | 151.5 | 31.4 | 245.7 |
| Stout moth | 4 | 2.2% | 22.5 | 19.5 | 6.4 | 45.8 |
| Swallowtail butterfly | 1 | 0.6% | 113.6 | nd | 113.6 | 113.6 |
| Tiger moth | 1 | 0.6% | 19.2 | nd | 19.2 | 19.2 |
| Carnivorous | **43** | **24.2%** | **442.3** | **1,497.7** | **1.2** | **7,393.6** |
| Bark centipede | 1 | 0.6% | 143.2 | nd | 143.2 | 143.2 |
| Carabid beetle | 2 | 1.1% | 177.9 | 154.5 | 68.6 | 287.2 |
| Cicada | 1 | 0.6% | 4.3 | nd | 4.3 | 4.3 |
| Fire colored beetle | 1 | 0.6% | 33.6 | nd | 33.6 | 33.6 |
| Firefly | 1 | 0.6% | 108.4 | nd | 108.4 | 108.4 |
| Flimy dome spider | 1 | 0.6% | 6.0 | nd | 6.0 | 6.0 |
| Ground beetle | 9 | 5.1% | 92.0 | 80.6 | 25.6 | 236.7 |
| Ground spider | 3 | 1.7% | 179.8 | 124.0 | 58.0 | 305.9 |
| Harvestman | 3 | 1.7% | 137.5 | 101.7 | 25.2 | 223.4 |
| Orb weaver spider | 1 | 0.6% | 84.5 | nd | 84.5 | 84.5 |
| Robber flies | 1 | 0.6% | 30.2 | nd | 30.2 | 30.2 |
| Scoliid wasp | 1 | 0.6% | 31.3 | nd | 31.3 | 31.3 |
| Scorpion (large) | 3 | 1.7% | 52.9 | 22.1 | 33.1 | 76.7 |
| Scorpion (small) | 2 | 1.1% | 75.7 | 73.0 | 24.1 | 127.3 |
| Scorpion (unspecified) | 1 | 0.6% | 240.6 | nd | 240.6 | 240.6 |
| Spider (mixed) | 6 | 3.4% | 2,498.1 | 3,589.9 | 1.2 | 7,393.6 |
| Stone centipede | 5 | 2.8% | 156.1 | 128.8 | 49.1 | 299.4 |
| Wolf spiders | 1 | 0.6% | 124.5 | nd | 124.5 | 124.5 |
| Omnivorous | **36** | **20.2%** | **467.4** | **1,342.3** | **15.6** | **7,301.9** |
| Amphibious litter cockroach | 8 | 4.5% | 535.9 | 1,186.2 | 67.0 | 3,469.4 |
| Arboreal/Tree snail | 2 | 1.1% | 165.1 | 47.6 | 131.4 | 198.8 |
| Burrowing cricket | 1 | 0.6% | 49.7 | nd | 49.7 | 49.7 |
| Bush cricket | 1 | 0.6% | 138.3 | nd | 138.3 | 138.3 |
| Camal cricket | 1 | 0.6% | 297.1 | nd | 297.1 | 297.1 |
| Dusky slug | 2 | 1.1% | 21.1 | 7.8 | 15.6 | 26.6 |
| Field cricket | 7 | 3.9% | 123.3 | 133.7 | 24.1 | 392.6 |
| Harvestman | 2 | 1.1% | 3,711.0 | 5,078.4 | 120.0 | 7,301.9 |
| Jerusalem cricket | 1 | 0.6% | 2,191.1 | nd | 2,191.1 | 2,191.1 |
| Land snail | 5 | 2.8% | 98.0 | 98.9 | 28.2 | 271.4 |
| Roundback slug | 5 | 2.8% | 130.6 | 148.0 | 38.1 | 391.8 |
| Tree cricket | 1 | 0.6% | 62.4 | nd | 62.4 | 62.4 |
| Coprophagous | **12** | **6.7%** | **60.0** | **42.0** | **6.0** | **144.0** |
| Dung beetle | 2 | 1.1% | 17.3 | 15.9 | 6.0 | 28.5 |
| Scarab beetle | 10 | 5.6% | 68.5 | 40.5 | 24.7 | 144.0 |
| Detritivorous | **29** | **16.3%** | **1,287.7** | **2,224.0** | **25.7** | **7,891.2** |
| American giant millipede | 10 | 5.6% | 403.5 | 346.0 | 87.9 | 1,059.7 |
| Ant | 1 | 0.6% | 489.0 | nd | 489.0 | 489.0 |
| Carrion beetle | 3 | 1.7% | 49.8 | 21.0 | 25.7 | 64.4 |
| Earthworm | 8 | 4.5% | 3,646.1 | 3,217.8 | 117.6 | 7,891.2 |
| Flat millipede | 3 | 1.7% | 1,019.5 | 899.3 | 82.7 | 1,875.9 |
| Termites | 1 | 0.6% | 42.9 | nd | 42.9 | 42.9 |
| Woodlice | 1 | 0.6% | 186.8 | nd | 186.8 | 186.8 |
| Woodroach | 2 | 1.1% | 106.3 | 48.9 | 71.7 | 140.9 |
| Grand Total | **178** | **100.0%** | **455.8** | **1,363.2** | **1.2** | **7,891.2** |

# Table S8 List of biota samples that were identified as outliers in their respective locations using the interquartile range (IQR) method and excluded from TMS and TMF linear regression analyses. [*Q1 and Q3 are the 25^th^ and 75^th^ percentiles, respectively].

| Location | Order | Common name | δ^13^C  (‰) | δ^15^N  (‰) | TL | Total Li  (ng/g) | Log[Li]  (ng/g) | *Q1 | *Q3 | IQR |
| --- | --- | --- | --- | --- | --- | --- | --- | --- | --- | --- |
| UMBS, US | Fresh litter | Plant litter | -29.4 | -1.9 | 1.4 | 560.01 | 2.75 | 26.56 | 152.36 | 125.76 |
| UMBS, US | Stylommatophora | Slug | -24.2 | -0.6 | 1.8 | 391.81 | 2.59 | 26.56 | 152.36 | 125.76 |
| UMBS, US | Arachnida | Spider | -25.9 | 6.5 | 3.8 | 612.31 | 2.79 | 26.56 | 152.36 | 125.76 |
| UMBS, US | Spirobolida | Millipede | -23.6 | -2.2 | 1.3 | 1,059.69 | 3.03 | 26.56 | 152.36 | 125.76 |
| UMBS, US | Opisthopora | Earthworm | -23.5 | 0.6 | 2.1 | 371.43 | 2.57 | 26.56 | 152.36 | 125.76 |
| UMBS, US | Opisthopora | Earthworm | -24.3 | 1.4 | 2.3 | 852.14 | 2.93 | 26.56 | 152.36 | 125.76 |
| Hubbard Brook, US | Fresh litter | Plant litter | -29.99 | -2.1 | 1.0 | 295.50 | 2.47 | 22.74 | 94.25 | 71.51 |
| Hubbard Brook, US | Lepidoptera | Moth | -29.1 | 3.2 | 2.6 | 223.99 | 2.35 | 22.74 | 94.25 | 71.51 |
| Angelo, US | Fresh litter | Plant litter | -29.5 | 3.9 | 2.4 | 4,496.72 | 3.65 | 45.44 | 971.51 | 926.07 |
| Angelo, US | Arachnida | Harvestman | -25.9 | 2.8 | 2.0 | 7,301.92 | 3.86 | 45.44 | 971.51 | 926.07 |
| Angelo, US | Arachnida | Spider | -25.0 | 5.4 | 2.8 | 7,393.56 | 3.87 | 45.44 | 971.51 | 926.07 |
| Angelo, US | Arachnida | Spider | -24.3 | 5.3 | 2.8 | 6,842.79 | 3.84 | 45.44 | 971.51 | 926.07 |
| Coweeta, US | Polydesmida | Millipede | -24.2 | 0.2 | 1.9 | 1,875.89 | 3.27 | 47.43 | 205.41 | 157.98 |
| Coweeta, US | Opisthopora | Earthworm | -25.3 | 2.0 | 2.4 | 7,891.16 | 3.90 | 47.43 | 205.41 | 157.98 |
| Tai Po Kau, HK | Old litter | Plant litter | -32.8 | -2.1 | 0.7 | 559.31 | 2.75 | 59.47 | 199.13 | 139.66 |
| Tai Po Kau, HK | Old litter | Plant litter | -32.5 | -1.5 | 0.9 | 709.99 | 2.85 | 59.47 | 199.13 | 139.66 |
| Tai Po Kau, HK | Orthoptera | Grasshopper | -31.2 | 0.8 | 1.6 | 1,623.13 | 3.21 | 59.47 | 199.13 | 139.66 |
| Tai Po Kau, HK | Hymenoptera | Ant | -27.3 | 2.9 | 2.2 | 488.96 | 2.69 | 59.47 | 199.13 | 139.66 |
| Shing Mun, HK | Old litter | Plant litter | -31.5 | 0.0 | 1.3 | 1,183.88 | 3.07 | 59.47 | 199.13 | 139.66 |
| Shing Mun, HK | Old litter | Plant litter | -31.3 | -0.8 | 1.1 | 1,304.53 | 3.12 | 59.47 | 199.13 | 139.66 |
| Shing Mun, HK | Blattodea | Cockroach | -28.2 | 0.4 | 1.4 | 3,469.40 | 3.54 | 59.47 | 199.13 | 139.66 |
| Shing Mun, HK | Opisthopora | Earthworm | -27.2 | 2.3 | 2.0 | 6,580.87 | 3.82 | 59.47 | 199.13 | 139.66 |
| Shing Mun, HK | Opisthopora | Earthworm | -26.0 | 4.8 | 2.7 | 6,629.43 | 3.82 | 59.47 | 199.13 | 139.66 |
| Shing Mun, HK | Opisthopora | Earthworm | -27.6 | -0.1 | 1.3 | 1,659.55 | 3.22 | 59.47 | 199.13 | 139.66 |
| Shing Mun, HK | Opisthopora | Earthworm | -27.5 | 3.0 | 2.2 | 5,066.89 | 3.71 | 59.47 | 199.13 | 139.66 |

# Table S9 List of plant litter and the 9 % of the invertebrates that accumulated greater than 1,000 ng/g of total Li concentrations on a dry weight basis (based on the old leaf litter threshold) from the US and HK forests (locations) sampled.

| Location | Order | Common name | Feeding habit | n | Total Li (ng/g) | Log[Li]  (ng/g) |
| --- | --- | --- | --- | --- | --- | --- |
| UMBS, US | Spirobolida | Millipede | Detritivorous | 0 | 1,059.69 | 3.03 |
| Angelo, US | Leaf litter | na | na | 1 | 4,496.72 | 3.65 |
| Angelo, US | Orthoptera | Cricket | Omnivorous | 4 | 2,191.06 | 3.34 |
| Angelo, US | Arachnida | Harvestman | Omnivorous | 32 | 7,301.92 | 3.86 |
| Angelo, US | Arachnida | Spider | Carnivorous | 95 | 7,393.56 | 3.87 |
| Angelo, US | Arachnida | Spider | Carnivorous | 84 | 6,842.79 | 3.84 |
| Angelo, US | Hemiptera | Plant bug | Herbivorous | 35 | 1,550.91 | 3.19 |
| Angelo, US | Polydesmida | Millipede | Detritivorous | 17 | 1,100.01 | 3.04 |
| Coweeta, US | Polydesmida | Millipede | Detritivorous | 5 | 1,875.89 | 3.27 |
| Coweeta, US | Opisthopora | Earthworm | Detritivorous | 1 | 7,891.16 | 3.89 |
| Tai Po Kau, HK | Orthoptera | Grasshopper | Herbivorous | 1 | 1,623.13 | 3.21 |
| Shing Mun, HK | Blattodea | Cockroach | Omnivorous | 2 | 3,469.40 | 3.54 |
| Shing Mun, HK | Opisthopora | Earthworm | Detritivorous | 2 | 6,580.87 | 3.82 |
| Shing Mun, HK | Opisthopora | Earthworm | Detritivorous | 2 | 6,629.43 | 3.82 |
| Shing Mun, HK | Opisthopora | Earthworm | Detritivorous | 2 | 1,659.55 | 3.22 |
| Shing Mun, HK | Opisthopora | Earthworm | Detritivorous | 2 | 5,066.89 | 3.71 |

# Table S10 The number of invertebrate composite samples based on order/family, analyzed and reported in the study (excluding those whose total Li concentrations were below the detection limits (2) and invertebrate larvae (2)) across all the forests studied from the US and Hong Kong.

#

| Invertebrate order | UMBS, US | Angelo, US | Coweeta, US | Hubbard Brook, US | Shing Mun, HK | Tai Po Kau, HK | Grand Total |
| --- | --- | --- | --- | --- | --- | --- | --- |
| Arachnida | 6 | 11 | 4 | 0 | 0 | 1 | **22** |
| Araneae | 0 | 0 | 0 | 1 | 0 | 0 | **1** |
| Blattodea | 0 | 0 | 2 | 0 | 9 | 0 | **11** |
| Coleoptera | 7 | 1 | 9 | 3 | 11 | 3 | **34** |
| Diptera | 1 | 0 | 0 | 0 | 0 | 0 | **1** |
| Hemiptera | 0 | 3 | 0 | 0 | 1 | 1 | **5** |
| Hymenoptera | 0 | 0 | 0 | 0 | 1 | 1 | **2** |
| Isopoda | 1 | 0 | 0 | 0 | 0 | 0 | **1** |
| Lepidoptera | 5 | 3 | 8 | 7 | 9 | 6 | **38** |
| Opisthopora | 2 | 0 | 2 | 0 | 4 | 0 | **8** |
| Orthoptera | 4 | 2 | 3 | 0 | 5 | 5 | **19** |
| Orthoptera | 0 | 1 | 0 | 0 | 0 | 0 | **1** |
| Phasmida | 0 | 0 | 0 | 0 | 0 | 1 | **1** |
| Polydesmida | 0 | 1 | 2 | 0 | 0 | 0 | **3** |
| Scolopendromorpha | 0 | 1 | 2 | 0 | 1 | 2 | **6** |
| Spirobolida | 3 | 2 | 5 | 0 | 0 | 0 | **10** |
| Stylommatophora | 5 | 1 | 5 | 1 | 2 | 1 | **15** |
| Grand Total | **34** | **26** | **42** | **12** | **43** | **21** | **178** |


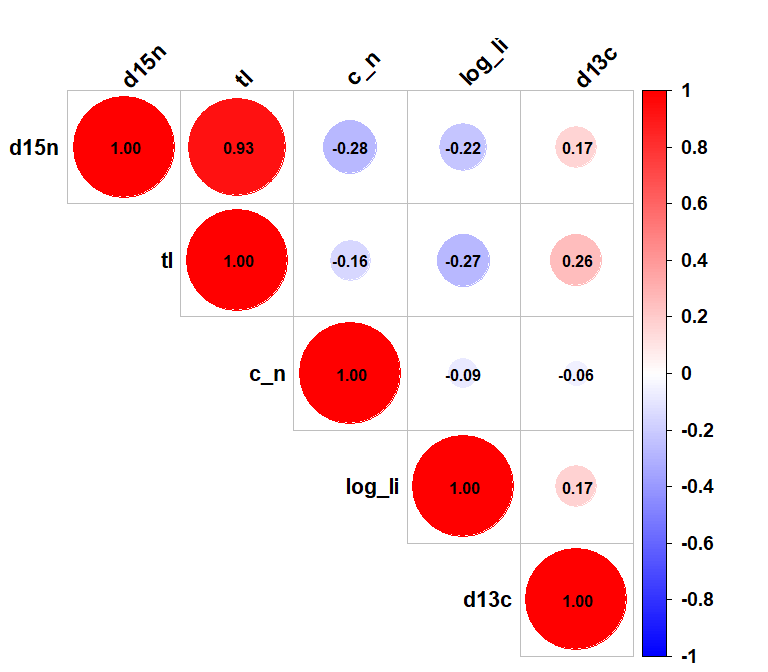


**Fig. S1.** Spearman correlation heatmap of log_10_-transformed Li *vs.* other parameters analyzed in the study.
